# Recent advances in nanotechnology for Parkinson’s disease: diagnosis, treatment, and future perspectives

**DOI:** 10.3389/fmed.2025.1535682

**Published:** 2025-01-22

**Authors:** Virendra Kumar Yadav, Seshathiri Dhanasekaran, Nisha Choudhary, Deepak Nathiya, Vishal Thakur, Rachna Gupta, Sheersha Pramanik, Pankaj Kumar, Nishant Gupta, Ashish Patel

**Affiliations:** ^1^Faculty of Sciences, Department of Microbiology, Marwadi University Research Center, Marwadi University, Rajkot, Gujarat, India; ^2^Department of Computer Science, UiT The Arctic University of Norway, Tromsø, Norway; ^3^Department of Life Sciences, Parul Institute of Applied Sciences, Parul University, Vadodara, India; ^4^Department of Pharmacy Practice, NIMS Institute of Pharmacy, NIMS University Rajasthan, Jaipur, India; ^5^Centre for Research Impact & Outcome, Chitkara University Institute of Engineering and Technology, Chitkara University, Rajpura, India; ^6^Department of Pharmaceutics, National Institute of Pharmaceutical Education and Research (NIPER), Ahmedabad, India; ^7^Department of Biotechnology, Bhupat and Jyoti Mehta School of Biosciences, Indian Institute of Technology Madras, Chennai, India; ^8^Department of Environmental Science, Parul Institute of Applied Sciences, Parul University, Vadodara, India; ^9^Department of Engineering and Medical Devices, River Engineering Pvt. Ltd., Greater Noida, India; ^10^Department of Life Sciences, Hemchandracharya North Gujarat University, Patan, India

**Keywords:** CRISPR/Cas9, dopaminergic pathways, gallium nanoparticles, neurodegenerative, neuroglia, Parkinson’s

## Abstract

Parkinson’s disease is a progressive neurodegenerative disease that destroys substantia nigra dopaminergic neurons, causing tremors, bradykinesia, rigidity, and postural instability. Current treatment approaches primarily focus on symptom management, employing pharmacological, non-pharmacological, and surgical methods. However, these treatments often result in fluctuating symptoms, side effects, and disease progression. Here, the authors have reviewed the emerging field of nanomedicine as a promising path for Parkinson’s disease treatment, emphasizing its potential to overcome the limitations of traditional therapies. Nanomedicine utilizes nanoparticles for targeted drug delivery, leveraging their small size and high surface area to volume ratio to cross the blood-brain barrier and deliver therapeutic agents directly to affected brain regions. Various nanoparticles, including lipid-based, polymeric, metallic, and carbon-based, have shown potential in Parkinson’s disease treatment. Additionally, nanocarrier systems like liposomes, nanogels, dendrimers, and solid lipid nanoparticles offer controlled and sustained release of therapeutic agents, enhancing their bioavailability and reducing side effects. This review provides insights into the pathophysiology of Parkinson’s disease, highlighting the mechanisms of neurodegeneration, the role of alpha-synuclein, and the disruption of dopaminergic pathways. It further discusses the application of gene therapy in conjunction with nanomedicine for targeted therapeutic interventions.

## 1 Introduction

Every year, a huge number of the population is affected by various neurological diseases like Alzheimer’s disease (1), Parkinson’s’ disease (PD), glioblastoma (2), different types of strokes (3), etc. The cases of such patients are continuously increasing worldwide, which could be attributed to the rapidly changing lifestyle, environmental factors, and genetic factors (4). Neurological disorders are disorders associated with the neurons and nervous system; one such disease affecting most of the population is PD (5, 6). PD is a progressive neurodegenerative disorder that is characterized by tremors, bradykinesia, rigidity, and postural instability and that mainly affects the motor system. PD significantly affects the quality of life (4, 7). PD mainly occurs due to the degeneration of dopamine-generating neurons in the substantia nigra. The substantial nigra a part of the brain which is vital for movement control. Due to the decline in dopamine levels in the brain, the communication between neurons gets disrupted, further leading to the hallmark motor symptoms of PD. Besides this, there are certain mon-motor symptoms, which include cognitive decline, mood disorders, and autonomic dysfunction, which also contribute to the disease burden. So, there is a requirement for precise diagnosis and treatment of PD (8, 9).

Currently, medical practitioners are focussing on symptom management rather than curing of the PD. Several conventional treatments, such as pharmacological, non-pharmacological, surgery, and emerging and experimental approaches, are available for treating PD (10). Among these, the pharmacological treatment-based approach is widely used to treat PD. The most extensively used pharmacological treatment is levodopa (11). Levodopa is a precursor to dopamine, which often combines with carbidopa to increase its effectiveness and reduce side effects. Besides this, dopamine agonists, monoamine oxidase B (MAO-B) inhibitors, and catechol-O-methyltransferase (COMT) inhibitors are some of the few options for the pharmacological treatment of PDs (12, 13). All three drugs aim to enhance or mimic dopamine activity in the brain. Moreover, surgical treatment involves deep brain stimulation (DBS) and lesioning surgery, out of which the former could be effective for patients of PD in the advanced stage who no longer respond adequately to the medicines. Even after treatment with such conventional approaches, the patients may experience fluctuating symptoms, side effects from the medicines, and disease progression (14, 15). So, there is a need for novel therapeutic strategies for PD that could overcome all the above-mentioned issues faced in conventional treatment. Several emerging novel techniques for treating PD include gene therapy (GT), nanomedicine, stem cells, and neuroprotective agents. Even though most of these techniques are in experimental stages only and need to go under clinical trials and evaluation, among all these emerging technologies, nanotechnology-based nanomedicines are gaining popularity among researchers for the theragnostic purposes of PD (16). Nanomedicine is the branch of nanosciences and nanotechnology that applies nanotechnology or nanoparticles (NPs) in medicine and offers promising new possibilities for diagnosing and treating PD (17). By developing the controlled size NPs, it is possible to deliver the drugs more effectively and precisely target affected regions in the brain. NPs are materials whose sizes fall from 1 to 100 nm (18, 19). Due to the small size and high surface area to volume ratio (SVR) of the NPs, they could easily cross the biological hurdles, like the blood-brain barrier (BBB) (20, 21). These BBBs traditionally restrict the efficacy of several treatments for neurological disorders. Furthermore, the NPs can be readily surface-modified with diverse organic compounds, ligands, and other agents to achieve target specificity, augmenting their efficacy. Additionally, certain NPs may function as nanocarriers, transporting drugs for PD on their surfaces, thereby facilitating the controlled and prolonged release of therapeutic agents. Furthermore, this focused drug administration should enhance therapeutic bioavailability and diminish side effects, thereby revolutionizing the management of PD (22, 23).

Recent theragnostic applications of nanomedicine for PD treatment include the formulation of various nanocarrier systems such as liposomes, nano gels, dendrimers, and solid lipid nanoparticles (SLNs) (24). All these nanocarrier systems are capable of encapsulating therapeutic agents for PD. The encapsulated therapeutic agents are shielded from enzymatic degradation, ensuring their delivery to the target site of action. Furthermore, nanomedicine presents opportunities for neuroprotective strategies by employing antioxidant and anti-inflammatory NPs to protect and repair damaged neurons. One more technique that demonstrates high possibilities for the treatment of PD is GT. GT involves the application of NPs as carriers for the delivery of desired genes that could correct or mitigate the underlying causes of PD (25). Besides this, integrating CRISPR/Cas9 technology with nanotechnology can address the root causes of neurodegeneration in PD. The integrated approaches significantly advance the quest for more effective and targeted therapies. The advancement of research in nanomedicine-based approaches promises to improve symptom management and patient outcomes and potentially slow the advancement of PD. The future of PD treatment lies in the successful translation of nanomedicine from the laboratory to clinical practice, opening the way for a new era in neurological care (25).

In the present review article, investigators have emphasized the basics of Parkinson’s disease, their pathophysiology, and current available treatment methods. Further emphasis was given to the drawbacks of the current drug for Parkinson’s disease. The authors have further focused on the role of nanotechnology, nanoparticles, and nanomedicine in treating Parkinson’s disease. The authors have highlighted the role of newly emerging techniques like gene therapy and CRISPR/Cas9 for the possible treatment of PD. Finally, emphasis was given to the possible integration of the nanoparticles with other emerging technologies for the symptomatic management of PD. Such investigation will pave the way for advanced technology and a new PD neurological care era.

## 2 Parkinson’s at a glance and their pathophysiology

PD is a chronic and progressive neurological disease that generally impacts the cortico-basal ganglia-thalamic circuitry in the brain (26). It is characterized by the degeneration of dopaminergic neurons in the substantia nigra, resulting in a deficiency of dopamine, a neurotransmitter vital for motor function (27). While PD is usually considered a disease that affects individuals over the age of 65, cases of early-onset Parkinson’s can also occur (28). Annually, it impacts approximately 3% of older people and ranks second behind Alzheimer’s disease (1). There is initial damage in β-oxidation in PD, reducing long-chain acylcarnitine levels (29). Consequently, there is a progressive accumulation of the presynaptic protein α-synuclein within intracellular fibres. The loss of dopamine neurons in the midbrain substantia nigra causes resting tremor, bradykinesia, and stiffness (30). The major problem is that no effective method exists to find the cause.

### 2.1 Pathophysiology of Parkinson’s disease

#### 2.1.1 Mechanism of neurodegeneration

PD is caused by the progressive degradation of dopaminergic nerve cells in the substantia nigra, a part of the brain that is essential for controlling movement (31). Dopamine levels rapidly decline due to neuronal death, disturbing the equilibrium between motor and non-motor functions. Furthermore, Lewy bodies and abnormal aggregates of the protein alpha-synuclein contribute to neuronal dysfunction and the clinical symptoms of the disease. A concise description of the pathogenesis of PD is provided. Initially, a significant advancement in comprehending PD involves elucidating the neurodegenerative process. The precise aetiology of this neuronal death is indeterminate; nonetheless, it is presumed to stem from the intricate interplay of genetic, environmental, and mitochondrial influences. Multiple critical pathways contribute to neurodegeneration in PD, oxidative stress (OS), mitochondrial failure, and neuroinflammation (32, 33).

OS is a prevalent phenomenon in the brain in PD, resulting from an imbalance between the production of deleterious chemicals known as reactive oxygen species (ROS) and the brain’s capacity to neutralize them (34). The increased OS in PD damages cellular components (lipids, proteins, and DNA), resulting in neuronal malfunction and death. This issue is further intensified by mitochondrial dysfunction, which impairs energy production and increases the creation of ROS. The characterization of neuroinflammation is carried out by the activation of microglia and the release of pro-inflammatory cytokines. The pro-inflammatory cytokines also play a crucial role in the neurodegenerative method, contributing to neuronal injury and cell death (35, 36).

#### 2.1.2 Role of alpha-synuclein

A small soluble protein (alpha-nuclein) is present in the brain in large amounts, mainly at the presynaptic terminals, and is expected to plays an important role in synaptic function and neurotransmitter release (37). Various pieces of literature have found that alpha-synuclein undergoes pathological aggregation in PD, forming insoluble fibrils. These insoluble fibrils accumulate as Lewy bodies and Lewy neurites within the nerve cells. The aggregation of such fibrils disrupts cellular function and contributes to neuronal death through several phenomena (38, 39).

Moreover, these proteins may misfold, further impair proteasomal and autophagic pathways. As a result, there will be an accumulation of damaged proteins and organelles. The synaptic function of the alpha-synuclein could also be disrupted by interfering with vesicle trafficking and the release of the neurotransmitter. Besides this, alpha-synuclein aggregates can spread from cell to cell, which may propagate pathology throughout the brain in a prion-like fashion. The presence of these aggregates is a hallmark of PD and is closely associated with the progression and symptomatology of the disease (38).

#### 2.1.3 Dopaminergic pathways

The dopaminergic pathways, mainly the nigrostriatal pathway, are critically involved in the motor symptoms of PD. Dopaminergic pathways consist of dopaminergic neurons that project from the substantia nigra pars compacta to the striatum, a brain area associated with coordinating movement (40). If these neurons are lost, there will be a significant lowering in dopamine levels in the striatum. As a result, there will be a disruption in the balance of excitatory and inhibitory signals needed for smooth and controlled movements. Dopamine is very important for modulating the activity of the basal ganglia. The basal ganglia are a group of nuclei that play a valuable role in motor control. Depleting the dopamine level in PD will lead to overactivity of the indirect pathway and under activity of the direct pathway within the basal ganglia circuitry (41). This imbalance increases inhibitory output to the thalamus and motor cortex. As a result, there will be characteristic motor symptoms of PD, like bradykinesia, rigidity, and tremors. The degeneration of dopaminergic neurons affects motor symptoms and non-motor functions. This is because dopaminergic pathways regulate mood, cognition, and autonomic functions. This explains the wide range of non-motor symptoms noticed in PD patients, including autonomic dysfunction, depression, anxiety, and cognitive impairment (42, 43).

**Figure 1:**
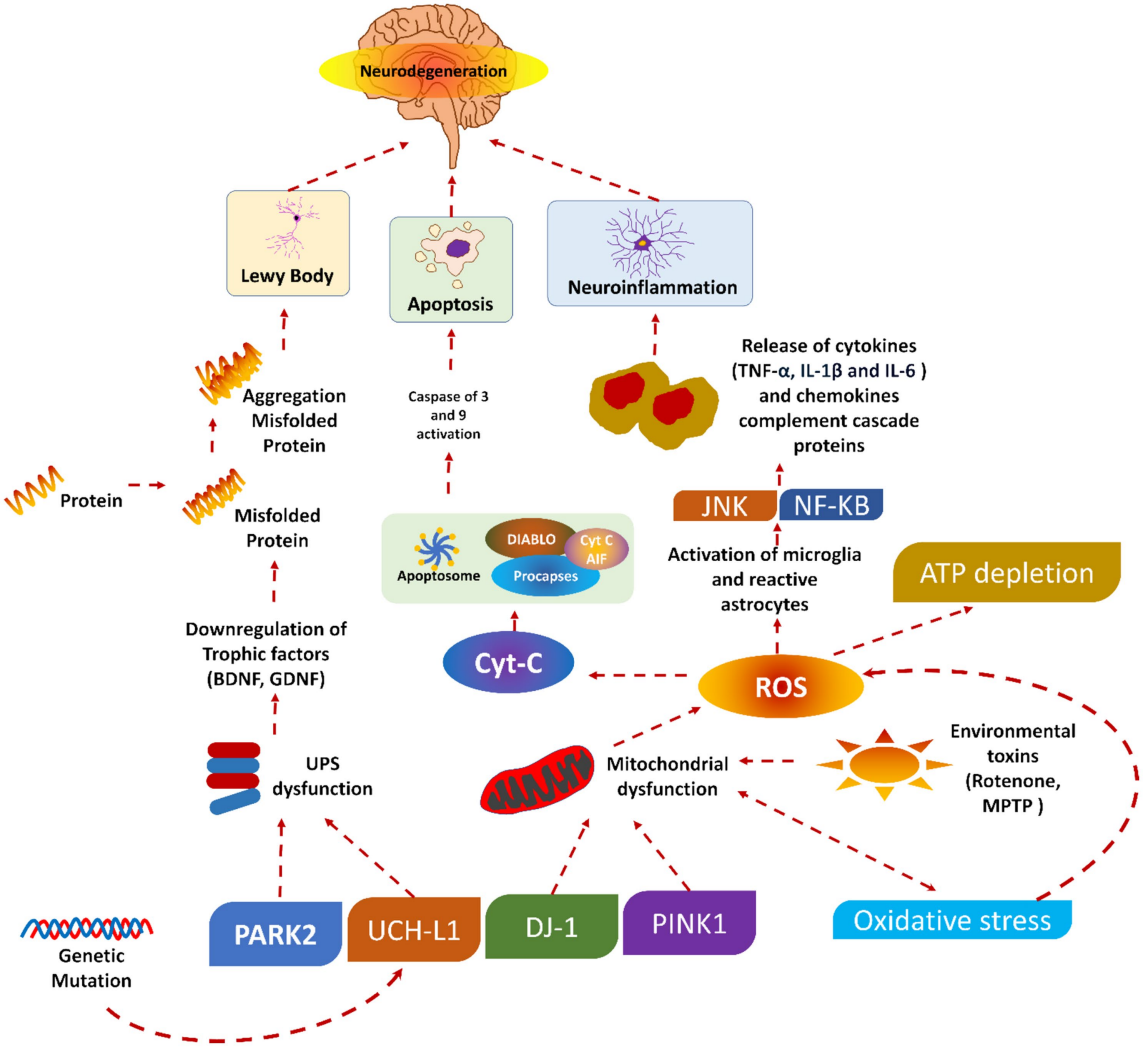
Pathophysiology of Parkinson’s diseases, reproduced from Doke et al. (189) with permission.

It could be concluded that the pathophysiology of PD involves the complex phenomenon of neurodegeneration, the pathological role of alpha-synuclein, and the disruption of dopaminergic pathways. Understanding these processes is vital for developing effective therapeutic strategies to slow or halt PD progression and improve patients’ quality of life. Figure 1 shows the pathophysiology of PD.

A summarized presentation of various genes involved in PD and their respective mechanisms of action is given in Table 1. Despite this, several pieces of information are still unknown, which warrants further investigation.

**Table 1:** Genes commonly implicated in the onset of Parkinson’s disease.

Gene	Mechanism of action	Dominant/recessive	References
*SNCA*	Engaged in protein degradation, membrane contacts, dopamine regulation, synaptic vesicle supply, autophagy, and mitochondrial dysfunction	Autosomal dominant PD	(192)
*PARK7*	Mutations in the DJ-1 gene cause gene expression loss. The animal models showed that the DJ-1 gene may be a neuroprotective redox sensor	Autosomal recessive familial PD	(193)
*PINK1*	Controls mitophagy, which eliminates damaged mitochondria. When mitochondria lose electrical charge, *PINK1* activates and phosphorylates ubiquitin at serine 65. A thorough analysis of the mechanisms reveals Parkin’s high-affinity binding, which phosphorylates *hPINK1* at *Ser65* in the N-terminal ubiquitin-like region. This activates E3 ligase, ubiquitylating the outer mitochondrial membrane substrate. Neuronal damage sources are unknown	Early onset recessive familial PD	(194)
*LRRK2*	PD mutations modify the kinase and GTPase activities. It enhances the process of adding phosphate groups to substrates and to itself through self-phosphorylation. The connection between neuronal injury is uncertain	Late-onset autosomal dominant familial PD	(195)
*PRKN*	Encodes RBR E3 ubiquitin-protein ligases. When this activity is lost due to mutation, it leads to the build-up of protein, mitophagy, and dysfunction in the mitochondria. The *PRKN* gene is named based on the consistent and predictable phenotypic outcomes it produces	Autosomal recessive juvenile PD (AR-JP)	(196)

## 3 Current treatment approaches for the PDs

**Figure 2:**
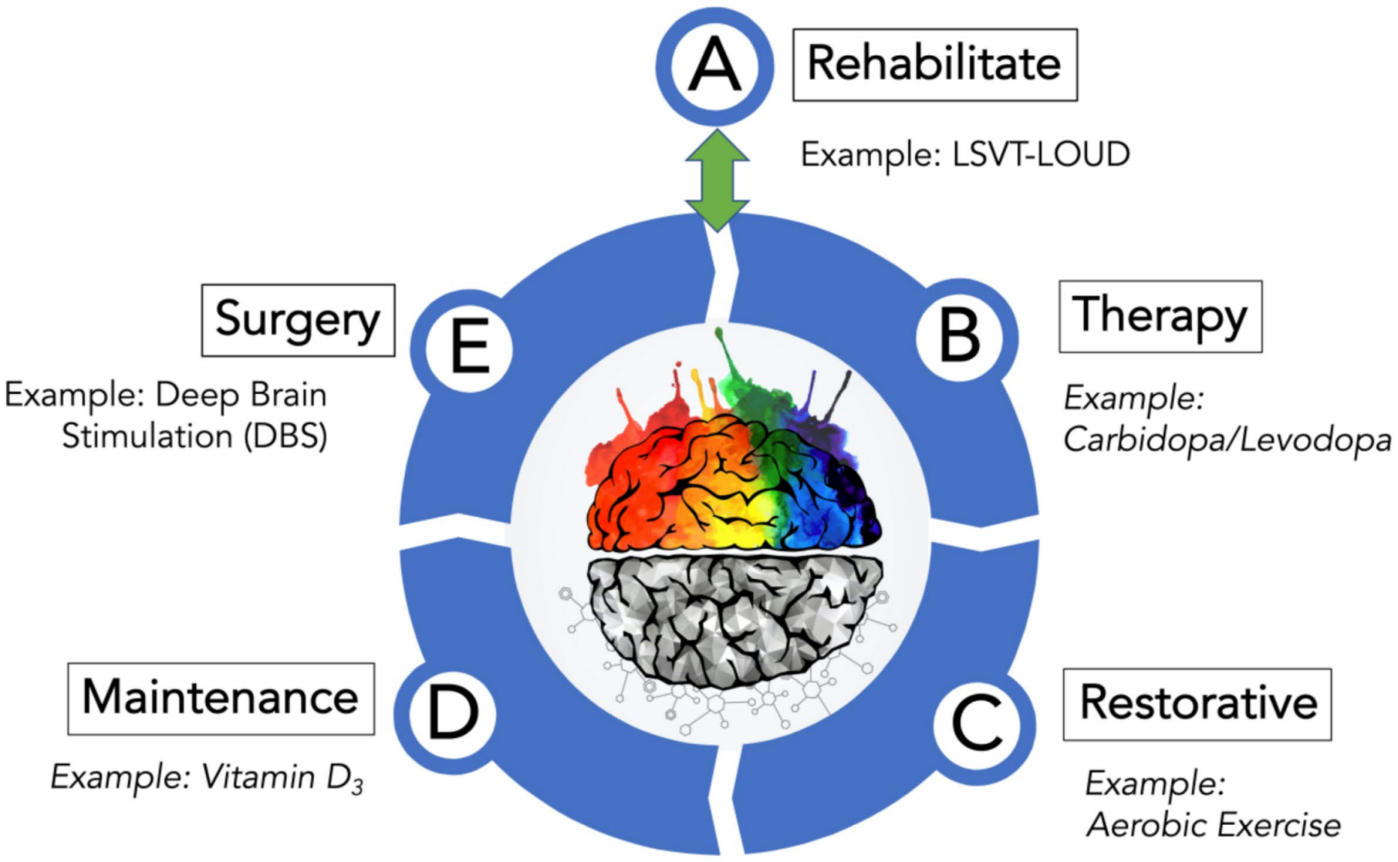
Treatment options for PD adapted from Church (190).

**Figure 3:**
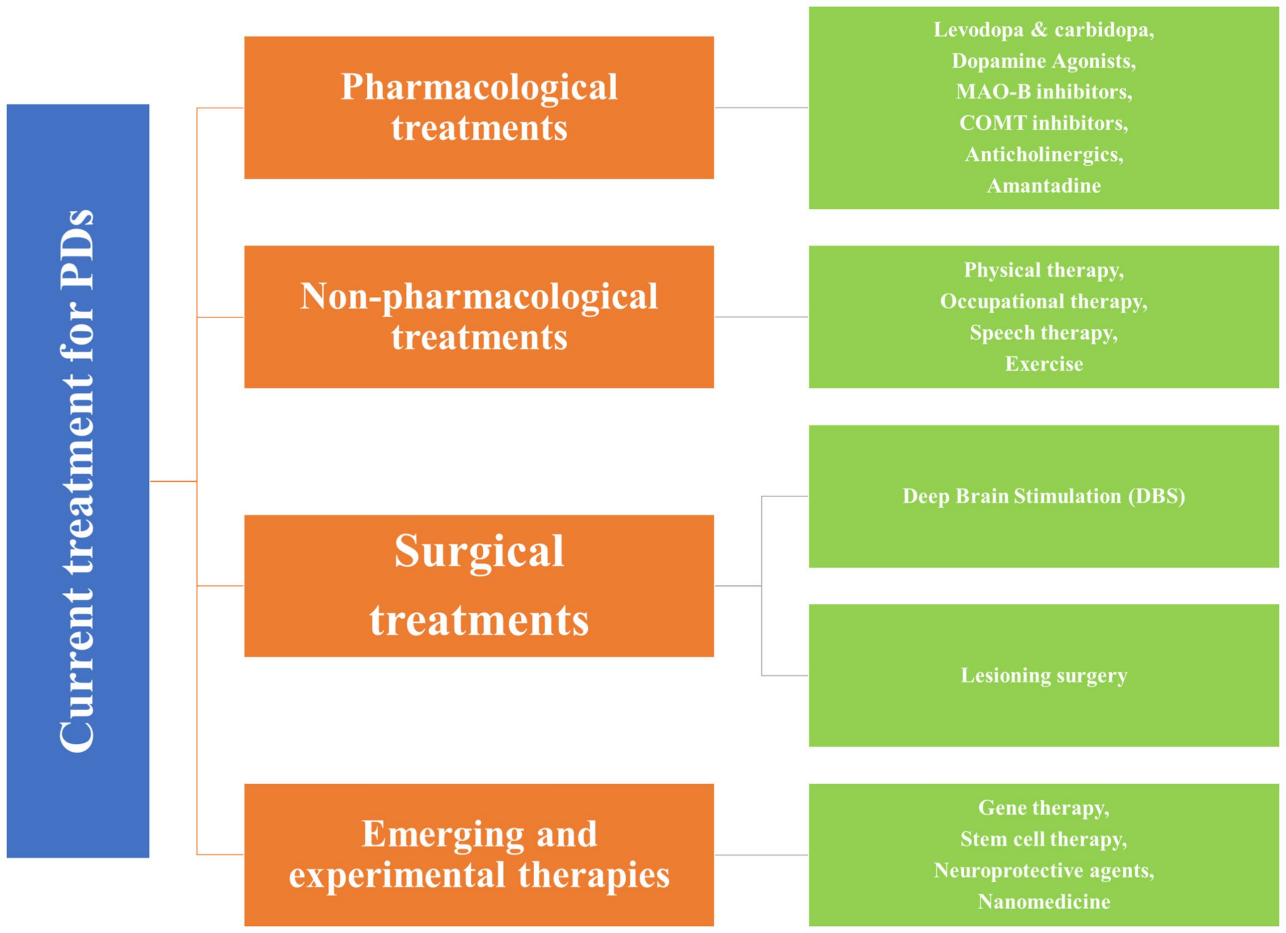
Current treatment approaches for the PDs.

**Figure 4:**
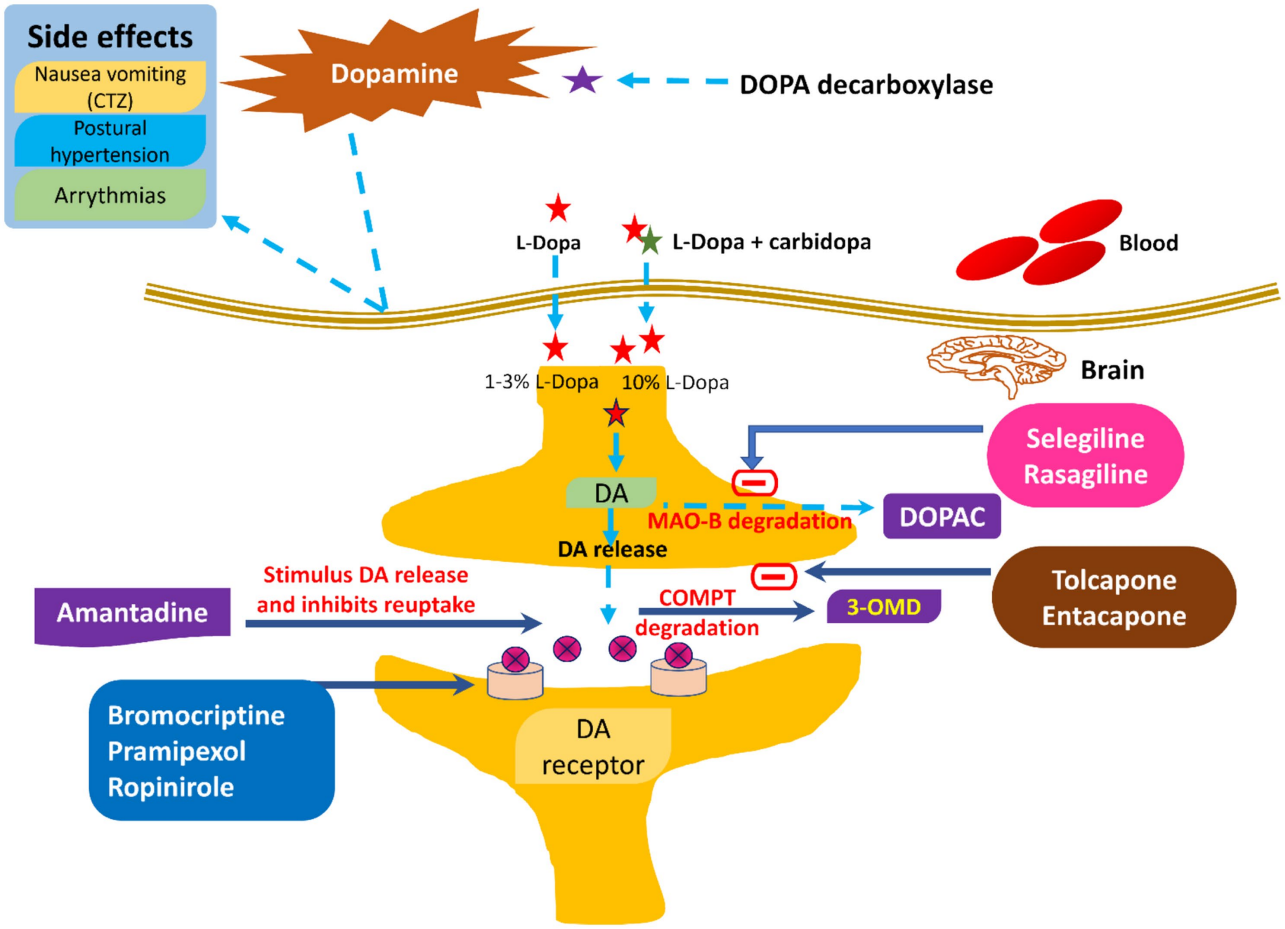
Current therapeutic management of PD along with their side effects.

Traditional approaches, such as pharmacological, non-pharmacological, and surgical, and some emerging treatment techniques, GT, stem cell therapy, neuroprotective agents, and nanomedicine, exist for treating PD (44). Figure 2 shows the broader categories of treatment options for PD, while Figures 3, 4 show various existing treatment strategies for PD.

### 3.1 Pharmacological treatments of PDs

Pharmacological treatments for PD encompass levodopa and carbidopa, dopamine agonists, MAO-B inhibitors, COMT inhibitors, anticholinergics, and amantadine (45). Levodopa is a highly effective and commonly utilized drug for PD, functioning as a precursor to dopamine that traverses the BBB and is subsequently converted to dopamine within the brain (46). Carbidopa is administered with levodopa to inhibit its early conversion to dopamine outside the central nervous system, thereby minimizing side effects like nausea and enhancing the quantity of levodopa penetrating the brain (47). The commonly utilized dopamine agonists are pramipexole, ropinirole, and rotigotine. These drugs replicate dopamine’s effects by directly activating dopamine receptors in the brain. These drugs may be utilized independently or in conjunction with levodopa to mitigate fluctuations in motor control. Selegiline and rasagiline are both inhibitors of MAO-B. MAO-B is an enzyme responsible for the degradation of dopamine in the brain, which subsequently leads to elevated dopamine levels and enhancement of motor symptoms (11, 48).

**Figure 5:**
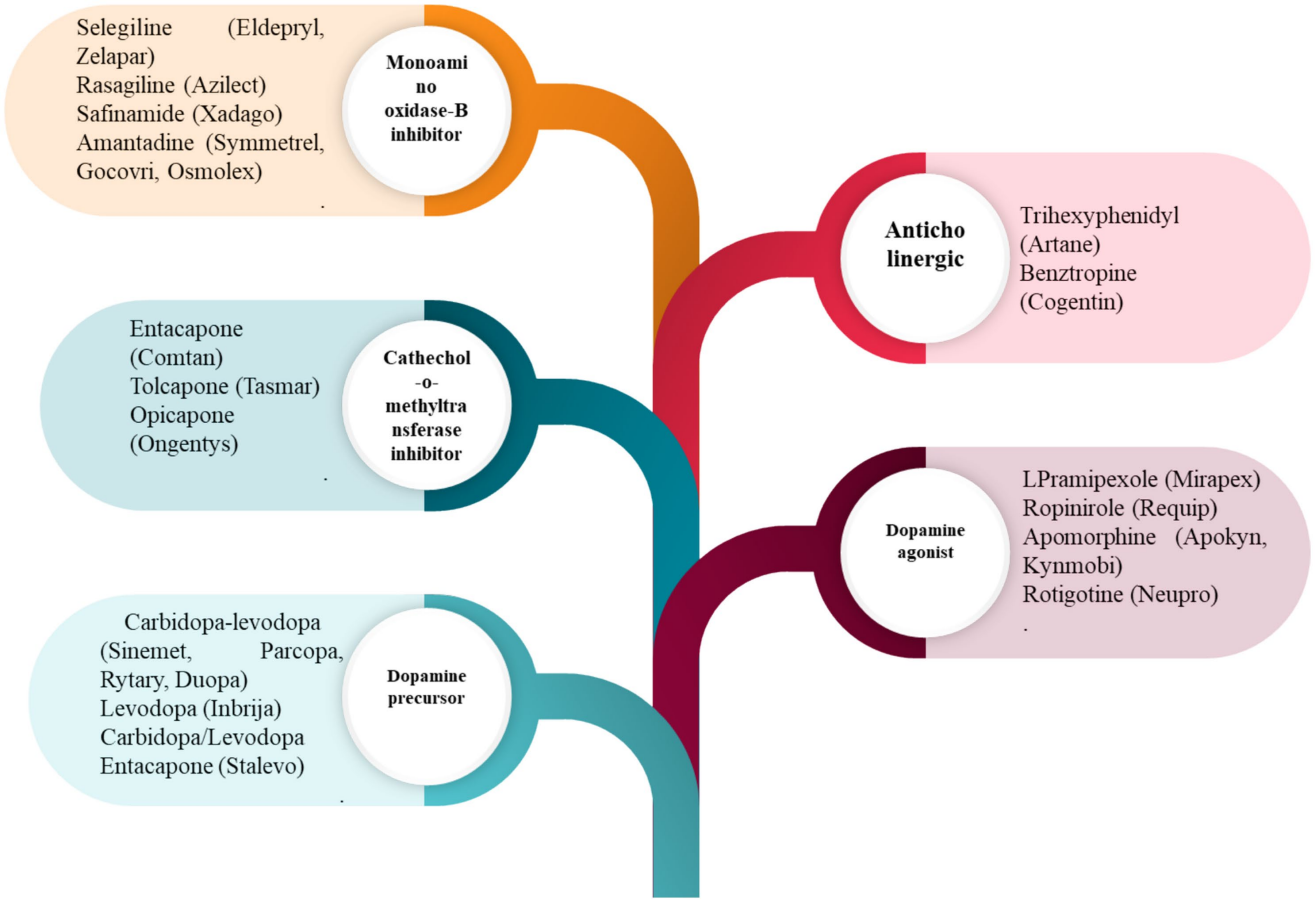
Common pharmacological drugs for treating symptoms of PD.

Both entacapone and tolcapone are COMT inhibitors that inhibit catechol-O-methyltransferase. COMT is an enzyme that breaks down levodopa (49). Besides this, COMT inhibitors are combined with levodopa to prolong its effects and reduce “off” periods. Both benztropine and trihexyphenidyl are anticholinergics that help control tremors and rigidity by decreasing acetylcholine activity (50). Acetylcholine is a neurotransmitter that can become overactive in PD due to the loss of dopamine (6, 51, 52). Amantadine is mainly an antiviral drug where the amantadine helps in reducing the symptoms of PD, particularly dyskinesia (involuntary movements) associated with prolonged use of levodopa. It could be concluded that there is little evidence to exhibit that dopamine prodrugs and levodopa would help alleviate dopamine neuron degeneration (28). It has been reported that such medical treatment causes several side effects like arrhythmia, gastrointestinal discomfort, extreme emotional changes, etc. (53). To date, not even a single drug is available, even after neuroprotective tests, which could delay or stop the progression of the disease. In addition to this, there are no effective biomarkers in PD, drugs having secondary hallucination and delusion effects, and the resistance of BBB. All these factors minimize the diagnosis and treatment of PD. Figure 5 shows various pharmacological treatment methods for PD (54).

Table 2 shows the classification of dopamine agonists, which are classified into two main classes: ergot-derived and non-ergot-derived. Among the ergot-derived dopamine agonists, bromocriptine, marketed as Parlodel, is administered orally. Cabergoline and lisuride are oral drugs in this category, though their market names are not specified. Pergolide, known as Permax, is another ergot-derived agent available in oral form (55, 56).

**Table 2:** Classification of dopamine agonists.

Ergot derived			Non-ergot derived		
Drug name	Market name	ROA	Drug name	Market name	ROA
Bromocriptine	Parlodel		Apomorphine	Apokyn	Subcutaneous
Cabergoline			Ropinirole	Requip	Oral
Lisuride		Oral	Rotigotine	NeuPro	Transdermal patch
Pergolide	Permax		Pramipexole	Mirapex	Oral

In contrast, the non-ergot-derived dopamine agonists include apomorphine, marketed as Apokyn, which is administered subcutaneously. Ropinirole, sold under the brand name requip, is an oral drug. Similarly, pramipexole, marketed as Mirapex, is also taken orally. Another notable non-ergot-derived agent is rotigotine, available as a transdermal patch under neupro (56, 57). This classification highlights the diversity of dopamine agonists in terms of their origin and routes of administration (ROA).

The pharmacological management of PD aims to enhance motor function by increasing dopamine levels. Additionally, there are limited treatments for non-motor symptoms, which can be significantly debilitating. Few treatments can impede or alter the progression of the disease. Levodopa and dopamine agonists are commonly utilized drugs for PD, although they may induce adverse consequences. Each patient’s treatment plan is personalized based on their symptoms, side effects, and requirements (43).

### 3.2 Non-pharmacological treatments of PDs

The non-pharmacological treatments of PDs include various approaches like physical, occupational, speech, and nutritional therapy and exercise (58, 59). Physical therapy focuses on improving mobility, balance, and strength through exercises, gait training, and assistive devices. Occupational therapy helps patients maintain independence by improving their ability to perform daily activities and recommending adaptive strategies and equipment (60). The difficulties associated with speech and swallowing could be addressed by speech therapy. Speech therapy provides exercises so that articulation, volume, and speech clarity, as well as techniques to manage dysphagia (difficulty swallowing), could be improved (61). In nutrition therapy, emphasis is given on the proper nutrition for managing symptoms and maintaining overall health. Advice from a well-trained dietitian could be very helpful for optimizing food choices. Moreover, a dietician could also help ensure adequate nutrient intake and manage gastrointestinal tract symptoms. Moreover, exercise could also help improve motor symptoms, balance, and overall well-being (62). The exercises may include regular physical activity like aerobic exercises, strength training, and flexibility exercises (63, 64).

**Figure 6:**
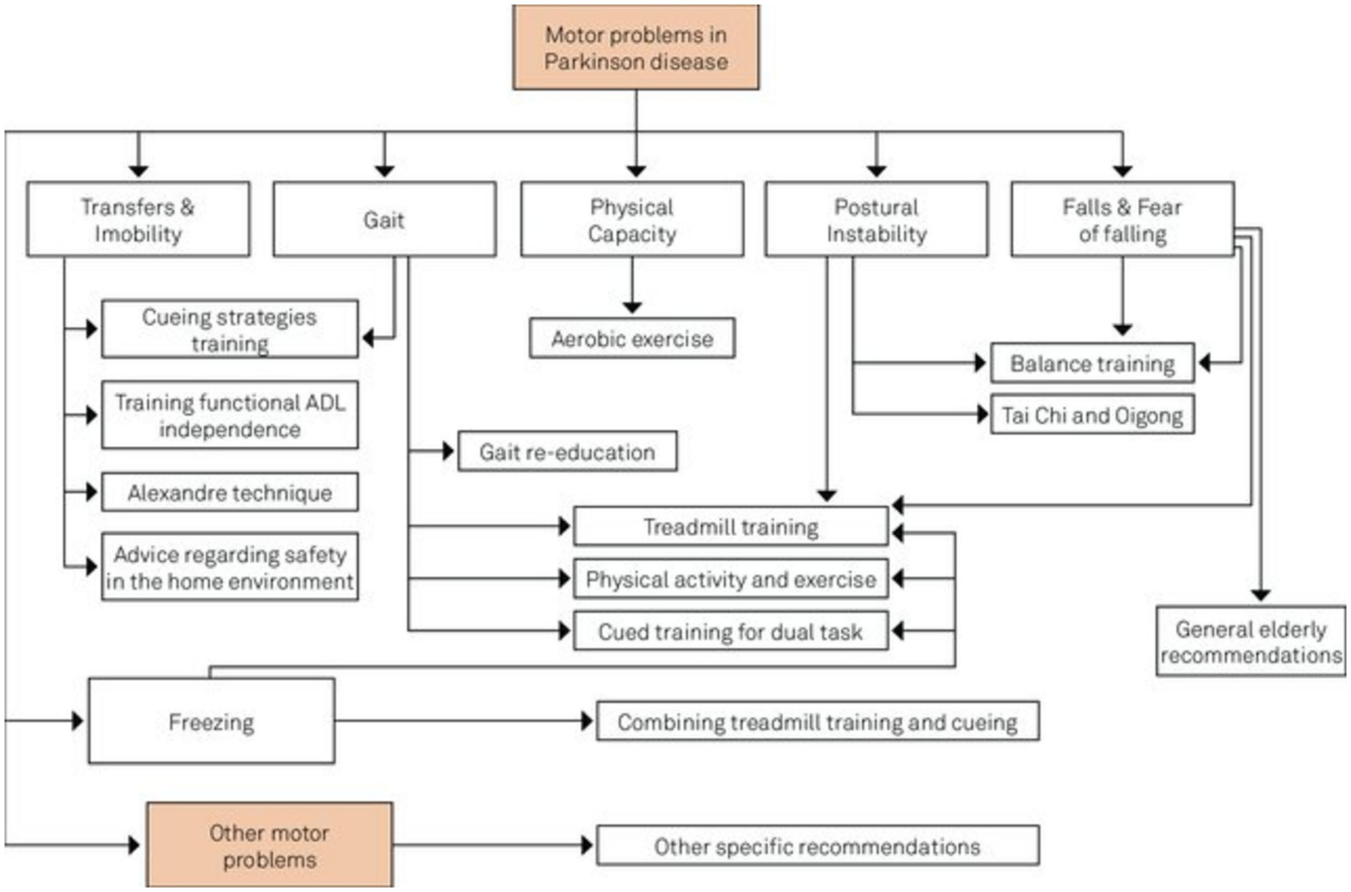
Intersection of non-pharmacological treatment interventions for PD adapted from Domingos et al. (191).

Kalbe et al. (65) recently emphasized the significance of non-pharmacological interventions in managing PD. These therapies are considered supportive or “add-on” strategies primarily designed to mitigate motor symptoms. Physiotherapy, speech-language, and occupational therapy have increasingly emerged as essential to comprehensive PD management. Additionally, non-pharmacological interventions such as cognitive training, cognitive behavioural therapy, and art or light therapy, previously deemed “exotic,” are now beginning to be integrated into therapeutic guidelines. Additionally, several advancements have been noted in this field, including (i) the expansion of intervention types, (ii) the standardization of intervention protocols, (iii) the development of digital intervention forms, (iv) the scientific evaluation of intervention feasibility and effects, (v) the understanding of underlying mechanisms of therapy-induced plasticity methods, (vi) the integration of non-pharmacological interventions into patient care concepts, and (vii) transition from merely symptomatic to preventive treatments (66). Figure 6 summarizes the various non-pharmacological treatments of PDs.

### 3.3 Surgical treatment of PD

PD could also be treated by using surgical therapy, which mainly includes DBS and lesioning surgery (67). DBS is a surgical procedure involving the implantation of electrodes into the subthalamic nucleus or globus pallidus internus of the brain. Additionally, these electrodes are linked to a pulse generator implanted in the thoracic cavity. Further, these pulse generators send electrical impulses to the brain to help regulate abnormal impulses. In lesioning surgery techniques (pallidotomy and thalamotomy), a small lesion is created in the targeted areas of the brain to disrupt abnormal brain activity (68). DBS is preferred over lesioning surgery from both available surgical therapies (14).

Sharma et al. (69) reviewed DBS and lesioning techniques for PD, addressing indications, selection processes, and management strategies. The investigators proposed that a team make the decision regarding surgical treatment for PD, taking into account the patient’s clinical characteristics, treatment efficacy, ease of use, associated risks, and overall quality of life. Still, DBS is the most preferred surgical technique, but there is a growing interest in new techniques like MR-guided focused ultrasound.

Recently, Foltynie et al. (70) presented the latest evidence in support of the optimal treatment of PD. Further investigators described an expert approach to several aspects of treatment choice with insufficient evidence base. Here, the investigators suggested treatment for different phases of PD. For instance, firstly, there should be initial treatment of motor PD; secondly, adjunctive treatment of motor PD; thirdly, non-oral therapies for the complex phase of PD; and finally, treatment of non-motor symptoms. The first treatment for PD should involve a detailed discussion about the diagnosis, treatment options, and how to maintain the patient’s independence and quality of life based on their specific symptoms. Some patients may not get enough relief from symptoms with initial treatment, and if certain symptoms persist, it may be necessary to consider other types of PD. Continuous dopaminergic or electrical stimulation targets the limited therapeutic range between symptom relief and peak dose dyskinesia, which arises during the advanced stages of PD. Non-motor symptoms in PD can affect many areas like mood, sleep, and pain and may be more challenging than movement issues, often linked to low dopamine levels and needing careful management.

### 3.4 Emerging and experimental approaches for the treatment of PDs

Above all, some emerging and experimental approaches for treating PDs include GT, stem cell therapy, neuroprotective agents, and nanomedicine (71). Literateurs have proven that in GT, genetic material has been introduced into the brain to correct or modify the underlying genetic causes of PD. Several clinical trials in their early stages explore using viral vectors to deliver genes. These genes may either increase the production of dopamine or protect nerve cells. Stem cell therapy is new to this field, and extensive research is being done on using these stem cells to replace lost dopaminergic neurons in the brain. Still, such approaches are in their experimental stages (72). Neuroprotective agents are also in their infancy, and several investigations are being conducted to formulate drugs that could help protect the neurons from degeneration. These neuroprotective agents include antioxidants, anti-inflammatory drugs, and agents targeting mitochondrial function (73). Besides this, nanomedicine is another technique in its infancy, but it has a huge potential for treating PDs. By utilizing nanotechnology, enhanced drug delivery and targeting could be achieved (25). Besides this, nanotechnology may improve efficacy and reduce the side effects of current treatment methods. Several nanocarriers (liposomes, nanoparticles) have been studied for their potential to cross the BBB and deliver therapeutic agents directly to affected brain regions. In contrast, Yuan et al. (74) explore the neurotoxic effects of SiO_2_ NPs, particularly in promoting PD-like pathology both *in vitro* and *in vivo*, suggesting a potential link between SiO_2_ NPs exposure and the initiation and progression of PD. While these emerging approaches hold promise, it’s important to acknowledge that they also have their limitations. This awareness is crucial for understanding the current challenges in PD treatment. Some of the complications associated with the current treatment approaches are summarized below in Table 3.

**Table 3:** Biological limitations and complications associated with the current treatment approaches for PD.

Therapy/drug	Drug name	Brand name	Biological limitations	Complications	References
Dopamine (DA)	Carbidopa/levodopa	Sinemet IR, Sinemet CR, Rytary, Duopa Inbrija	Characterized by hydrophilicity, Inability to traverse the BBB, limited plasma half-life, and low bioavailability	Nausea, dyskinesia, drowsiness	(197, 198)
Dopamine agonist	Apomorphine Pramipexole Ropinirole Rotigotine	Apokyn, Kynmobi Mirapex, Mirapex ER Requip, Requip XL Neupro	Poor pharmacokinetic profile and inadequate BAV	Sedation, visual hallucinations, euphoria, psychosis, delirium	(76, 77, 199)
Amantadine		Gocovri	Primarily really cleared. Close monitor kidney impairment and avoid it in severe cases	Decreased concentration, fatigue, euphoria, psychosis	(75)
COMT inhibitors (entacapone)	Opicapone Entacapone Carbidopa, levodopa, and entacapone Tolcapone	Ongentys Comtan Stalevo Tasmar	Poor pharmacokinetic profile, inability to cross the BBB, and poor BAV	Psychosis, abnormal disturbances, agitation	(78, 199)
L-dopa		Sinemet® Madopar	Short half-life, insufficient pharmacokinetic profile, and BAV (less than 1% can penetrate the brain milieu)	Depression, visual hallucinations, hypomania, abnormal sleep and dreams, psychosis, cognitive impairment	(79, 80)
MAO-B inhibitors (selegiline)	Selegiline Rasagiline Safinamide	Elderpryl, Zelapar Azilect Xadago	Poor pharmacokinetic profile, inability to cross the BBB, and poor BAV	Psychosis, abnormal sleep, agitation	(81, 82)
Anticholinergics	Trihexyphenidyl	Cogentin	No direct action on the dopaminergic system; their effects are limited to acetylcholine (muscarinic) receptors	Constipation, urinary retention, hallucinations, drowsiness, tachycardia, confusion, blurred vision, dementia, cognitive deficits, and related symptoms	(200–203)
	Benzhexol (trihexyphenidyl) benztropine mesylate	Cogentin	Limited efficacy over time	Agitation, insomnia, delirium, anxiety, psychosis, visual hallucinations	(83)
Adenosine A2A receptor antagonist		Nourianz Istradefylline	Eliminated only through hepatic metabolism. Monitor closely in situations of hepatic impairment and avoid severe instances	Insomnia, nausea, dizziness, constipation, hallucination, and dyskinesia	(204)

From the above table, it could be concluded that even though each drug is beneficial in the treatment of PDs, it also has several complications. For instance, amantadine may lead to decreased concentration and fatigue, along with the possibility of euphoria and psychosis (75). Dopamine agonists exhibit efficacy in managing PD symptoms but can be associated with sedation, visual hallucinations, euphoria, and psychosis, potentially leading to delirium (76, 77). COMT inhibitors like entacapone may induce psychosis, abnormal disturbances, and agitation (78). L-dopa, a cornerstone in PD treatment, may lead to depression, visual hallucinations, hypomania, abnormal sleep patterns, psychosis, and cognitive impairment (79, 80). Similarly, MAO-B inhibitors (selegiline) can lead to psychosis, abnormal sleep, and agitation (81, 82). Finally, benzhexol may induce agitation, insomnia, delirium, anxiety, and visual hallucinations (83). In clinical practice, weighing the therapeutic benefits against the potential adverse effects is crucial in individualizing treatment approaches for patients with PD.

## 4 Nanoparticles for the treatment of PD

**Figure 7:**
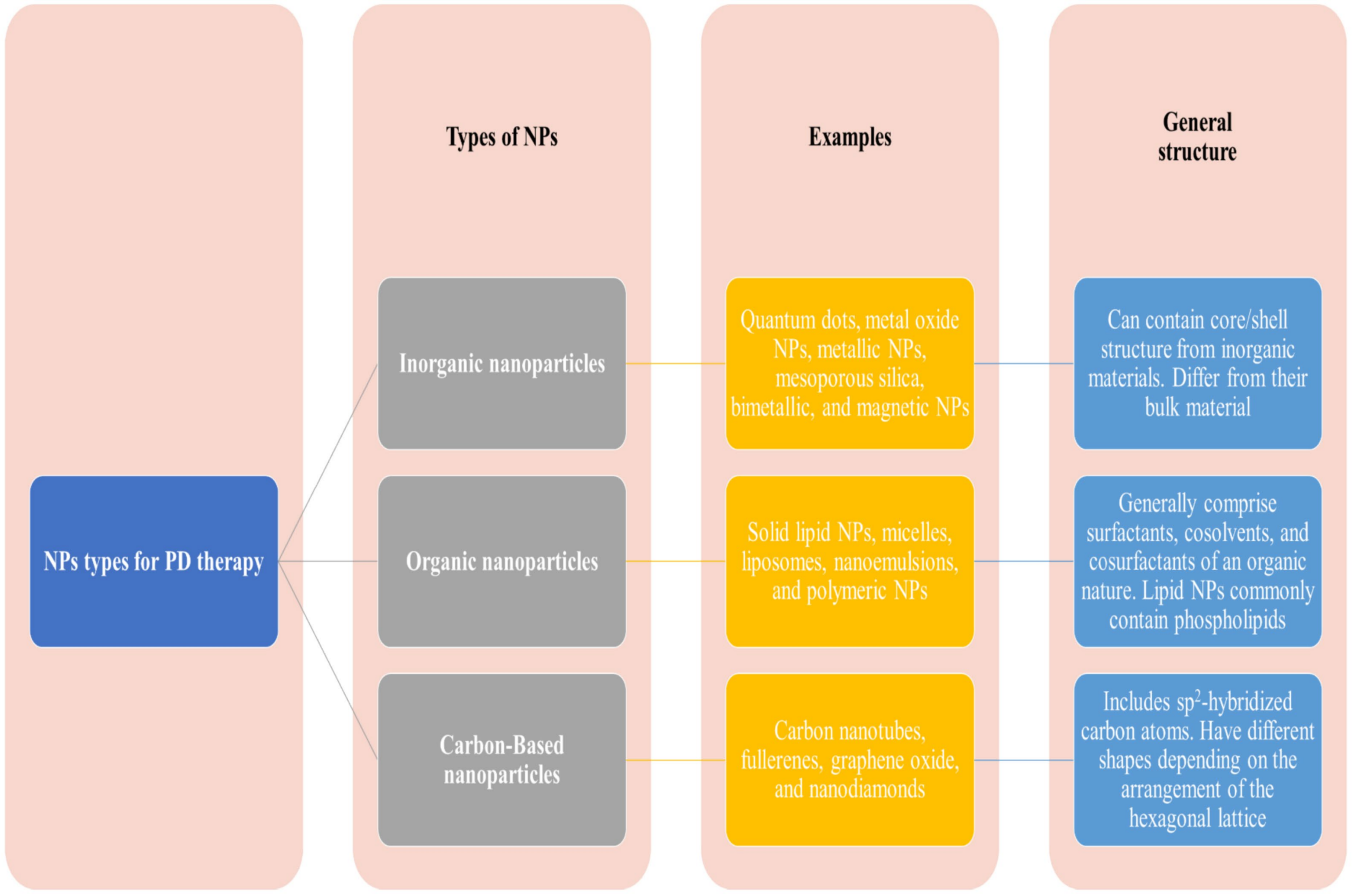
Major classes of nanoparticles used in nanomedicine.

NPs offer a promising approach to treating PD due to their small size. Besides this, the NPs could be easily engineered for specific targeting and the potential to improve drug bioavailability and efficacy (84). The NPs could be of various shapes, sizes, and origins, i.e., metallic or organic, solid or liquid, and carbon-based. The widely used NPs for treating PDs are lipid-based NPs, polymeric NPs, metal NPs, and carbon-based NPs, described below in detail. NMs hold substantial promise for progressing diagnostics in PD through three vital strategies (85, 86). Firstly, NMs offer the potential for governing crucial biomarkers such as α-synuclein, a protein associated with PD pathology (87). By preparing nanoscale tools and sensors, the detection and quantification of α-synuclein levels can be improved, assisting early diagnosis and disease monitoring. Secondly, NMs enable the monitoring of active dopamine neurons, providing beneficial insights into the status of dopaminergic neurotransmission, which is central to PD. NMs are efficient carriers for delivering therapeutic agents to specific target regions in the brain. The current NMs demonstrate effectiveness by addressing dopamine deficiency, caspase-3 activation, α-synuclein accumulation, OS, mitochondrial dysfunction, inflammation, and growth factor supplementation (88). The findings indicate a broad spectrum of potential approaches for Parkinson’s disease therapy (89). Generally, NPs can be classified as inorganic, organic, or carbon-based NPs (Figure 7).

### 4.1 Lipid-based nanoparticles

**Figure 8:**
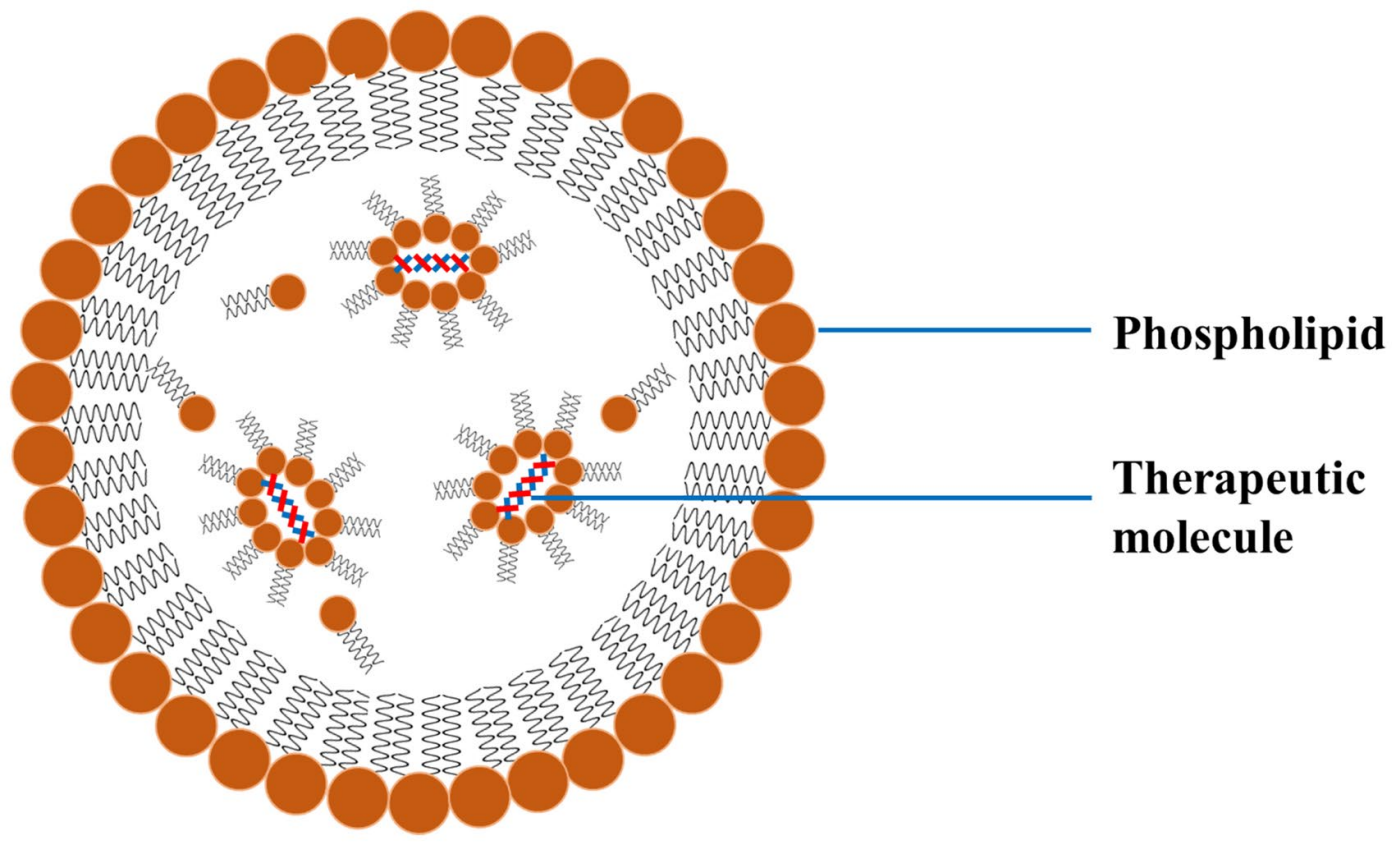
A schematic diagram of LNP exhibiting the outer phospholipid layer and the encapsulated therapeutics reproduced from Jagaran et al. (145) with permission.

Lipid-based NPs mainly comprise liposomes and SLNs, which are commonly investigated for treating PD. Such NPs encapsulate hydrophilic and hydrophobic drugs, protect them from degradation, and enhance their delivery across the BBB (90). Liposomes are spherical vesicles made up of lipid bilayers and are particularly advantageous due to their biocompatible nature and ability to fuse with cell membranes (Figure 8). The fusion of the liposomes with the cell membrane facilitates the release of the drug directly into the target cells. SLNs are made up of solid lipids, which offer a stable alternative to liposomes and can be applied for the effective delivery of lipophilic drugs effectively (91).

### 4.2 Polymeric nanoparticles

Polymeric NPs are organic compounds composed of biocompatible and biodegradable polymers like polylactic acid (PLA) and polyglycolic acid (PGA) (92). These polymeric NPs form an important class of NPs extensively applied for treating PD (92). One major advantage of using these NPs in PD treatment is their ability to release drugs in a controlled and sustained manner. This reduces dosing frequency and makes it easier for patients to stick to their treatment plan. These polymeric NPs constitute an important class of NPs that are widely used in the treatment of PD (93). One major advantage of using these NPs in PD treatment is their controlled and sustained drug release, which reduces dosing frequency and improves patient compliance (94, 95). Polymeric NPs are designed so that they get dissolved by themselves within the body. Due to the degradation of such polymeric NPs, the encapsulated drug is released over a predetermined period. Besides this, such polymeric NPs could also be functionalized with targeting ligands to enhance the specificity of dopaminergic neurons (96).

Various studies have investigated the administration of exogenous dopamine (DA) and L-dopa into the CNS to supplement the reduced levels of endogenous DA and counteract the loss of dopaminergic neurons. Advanced polymeric nanoparticulate formulations can potentially improve PD symptoms by delivering DA directly to the brain (97).

Arisoy et al. (12) developed a NP system using PLGA and WGA to deliver L-dopa through the nose, which helps the drug reach the brain directly and avoids the BBB. This method showed better drug delivery to the brain with fewer side effects than traditional oral or nasal methods, as seen in tests on mice with PD. The NPs system had a high drug retention rate of 73% and released L-dopa over 9 h, indicating its effectiveness and safety in the nasal cavity. However, using this delivery method may be difficult for patients with nasal issues like sinusitis or rhinitis, which could affect how the system works.

Ragusa et al. (98) effectively synthesized chitosan NPs that contained DA and evaluated the neuroprotective properties of the system *in vitro* using human neuroblastoma SH-SY5Y cells. They conducted additional research on the diverse impacts of DA, uncoated chitosan NPs, and chitosan-dopamine NPs in generating ROS. Monge-Fuentes et al. (99) created a new NP system loaded with DA (7 μg DA per mg of NPs) made up of albumin and PLGA (353 to 497 nm in diameter; PDI: 0.4 to 0.6; zeta potential −27 to −37 mV) that helps improve motor skills in mice with Parkinson’s disease by delivering dopamine and crossing the BBB effectively.

Vong et al. (100) developed a nano-polymeric drug system (NanoDOPA) to enhance the brain delivery and therapeutic efficacy of L-dopa in a mouse model of PD. The drug gradually released L-dopa through chymotrypsin degradation and significantly enhanced the *in vivo* pharmacokinetic profile of L-dopa in comparison to free L-dopa. The administration of NanoDOPA in the MPTP-induced PD mouse model led to significant enhancements in motor symptoms and reduced dyskinesia commonly linked to L-dopa treatment (see Table 4).

**Table 4:** The utilization of polymeric nanoparticulate systems to replace DA/L-dopa in PD.

Polymeric nanoparticulate system	Therapeutic agent loaded	ROA	Particle size (nm)	Model/cell-line	Remarks	References
PLGA/WGA	L-dopa	Intranasal	652.0 ± 67.7	MPTP-injected mice	Compared to traditional oral or intranasal L-dopa, there is a reduction in cytotoxicity, an increase in therapeutic efficacy, and a decreased side effects	(12)
PLGA	DA	Intravenous	120	6-OHDA-injected rats	Compared to free DA: higher plasma concentration and longer half-life. Significant reduction in ROS autoxidation, dopaminergic neuronal death, and D2 receptor hypersensitivity. Fewer side effects	(205, 206)
PLGA/albumin	DA	Intraperitoneal	353 to 497	6-OHDA-injected mice	Compared to free L-dopa: substantial improvements in motor symptoms	(99)
PEG-*b*-poly(L-dopa(OAc)_2_)	L-dopa	Intraperitoneal	∼52.2	MPTP-injected mice	Compared to free L-dopa: sustained release excellent *in vivo* pharmacokinetic profile enhancement	(100)
					Higher AUC and plasma levels	
					Significant motor symptom improvement and dyskinesia suppression	
					Lower cytotoxicity	
Self-assembled neuromelanin-inspired polymer/BIX/Fe(AcO)_2_	DA	Intracerebroventricular (i.c.v.) injection/intranasal	(56 to 64) ± 9.0	Healthy adult male Sprague–Dawley rats/6-OHDA-injected rats	Compared to free DA	(207)

### 4.3 Metallic nanoparticles

Metallic NPs (Ag, Au, Zn, Pt, Fe, etc.) also play a crucial role in treating PD due to their small size and unique optical and electronic properties (101, 102). Due to these features, such NPs could be exploited for therapeutic and diagnostic purposes in PD. The various metallic NPs used to treat PDs are gold (AuNPs) and silver nanoparticles (AgNPs) (103). For instance, AuNPs could be functionalized with drugs or targeting molecules and used for targeted delivery of drugs or as contrast agents for imaging. Besides this, such metallic NPs can be used to modulate the activity of enzymes engaged in OS. The OS is a key pathological PD property, offering neuroprotective effects (104, 105). Among metallic NPs, noble NPs like gold and silver have gained huge attention due to their high stability, easy synthesis routes, and easy surface functionalization.

AuNPs and AgNPs have demonstrated promise in inhibiting the aggregation of extracellular and intracellular protein aggregates in neurodegenerative disorders. Both the NPs can function as stabilizers by selectively attaching to certain areas of protein molecules, thereby preventing protein misfolding (106). This interaction hampers the proteins from assuming abnormal structures, creating harmful clusters. Furthermore, these noble NPs can act as “seeds” for protein aggregation. By emulating the protein’s original form, they entice the misfolded proteins and assist in their attachment to the NP surface. This process isolates the proteins from other molecules, decreasing their capacity to combine and create larger clusters. Moreover, both the NPs have exceptional catalytic capabilities. Both can facilitate the breakdown of misfolded proteins by catalytic mechanisms, such as the generation of ROS or the stimulation of cellular enzymes responsible for removing proteins. This augmented protein breakdown helps in preventing the buildup of harmful protein aggregates. The well-surface functionalized NPs can specifically engage with inflammatory cells in the brain, thus reducing the neuroinflammatory response. Due to the constant inflammation in neurodegenerative diseases, AuNPs and AgNPs have shown inherent antioxidative characteristics. Both of these NPs can eliminate ROS, which is known to play a significant role in causing neuronal damage through neuroinflammation (106). These NPs may improve brain inflammation caused by OS by decreasing levels of ROS. Moreover, it has been evident from the literature that both noble NPs might impede the synthesis and discharge of proinflammatory cytokines, including interleukin-1β (IL-1β), tumor necrosis factor-alpha (TNF-α), and IL-6. These cytokines play a vigorous role in neuroinflammation. NPs can potentially decrease the inflammatory cascade in the brain by inhibiting these cytokines. Furthermore, AuNPs and AgNPs regulate the nuclear factor-kappa B (NF-κB) pathway. NF-κB is a crucial transcription factor that activates many proinflammatory genes. Nps disrupt the NF-κB signalling pathway, inhibiting the synthesis of inflammatory mediators (104, 107).

AuNPs, as metallic NPs, are the most widely used in medicine due to their chemically inert nature, as in most cases, they do not undergo oxidation processes (108). This unique property makes them versatile and applicable in various medical contexts. Due to this reason, the surface of AuNPs can be easily functionalized with various types of chemical groups or dyes. Literature proves that AuNPs can act as a contrast and therapeutic agent in CNS disorders, further highlighting their potential in diverse medical applications (108, 109).

Hu et al. (110) developed composite materials of AuNPs (CTS@GNP-pDNA-NGF) by combining electrostatic adsorption and photochemical immobilization methods. These composites can potentially carry plasmid DNA (pDNA) and selectively target particular cell types. The Au NPs were introduced into cells through endocytosis to suppress the death of PC12 cells and dopaminergic neurons. Further, Au NPs composites are utilised in *in vivo* PD models, where they may effectively penetrate the BBB and accumulate in the brains of mice. It is concluded that the AuNP composites exhibit favourable therapeutic effects for *in vitro* and *in vivo* models of PDs.

AuNPs have also been employed in PD models. For instance, a study led by da Silva Córneo et al. (111) demonstrated that administering AuNPs to a mouse model of PD produced by reserpine significantly reduced OS markers and improved motor symptoms. The administration of AuNPs resulted in a partial improvement of neurotrophic factors. Specifically, the therapy at a concentration of 20 nm with a dosage of 2.5 mg/L successfully reversed the symptoms of the generated PD without causing any harmful effects.

A study by Xue et al. (112) exhibited that AuNPs synthesized from the root extract of *Paeonia moutan* effectively remove ROS and reduce the levels of inflammatory cytokines in microglial cells, as demonstrated *in vitro*. Moreover, the *in vivo* findings confirmed that therapy with AuNPs effectively avoided neuroinflammation in dopaminergic neurons and elevated dopamine levels. As a result, the mice with PD were protected from motor abnormalities. Both studies showed that therapy with AuNPs reversed the behavioural symptoms caused by PD, indicating the effective protective impact of AuNPs on dopaminergic neurons without any toxic effects. This treatment also prevented side symptoms associated with the condition.

The compatibility and interaction of AuNPs with pharmacological drugs render anti-inflammatory and other targeted drugs significant. These drugs are utilized in the treatment of neurodegenerative diseases, such as PD and AD. A study demonstrated that L-dopa, when associated with AuNPs, can mitigate the adverse effects of the drug while enhancing its therapeutic efficacy and prolonging its effect duration without compromising potency. The potential applications of Au NPs are effective and promising, broadening the scope of NP utilization in biomedical contexts as a novel treatment (113). Besides these two noble metal NPs, several other metal oxide NPs (iron oxide and silica) have also been used as therapeutic and contrast agents for PDs.

### 4.4 Carbon-based nanoparticles

Besides polymeric and metallic NPs, certain carbon-based NPs are widely used to treat PD. These carbon-based NPs include carbon nanotubes (CNTs), fullerenes, and graphene. The CNTs could be either single-walled (SWCNTs) or multi-walled (MWCNTs) (114). All these carbon-based NPs have high surface area, high mechanical strength, and the ability to penetrate cell membranes, which hold a promising potential for treating PD. Moreover, the CNTs could be further surface functionalized with various molecules to increase their solubility and biocompatibility. Surface-functionalized and biocompatible CNTs could be used to target the delivery of therapeutic agents (115). Both graphene and its derivatives have excellent conductivity and flexibility, which could be used to deliver drugs. Besides this, graphene and its derivatives act as scaffolds for neuronal growth, potentially facilitating the repair of damaged neural tissues (116).

One study used molecular dynamics simulation tools to investigate the impact of 4 forms of last-generation graphene-based nanostructures on preventing α-synuclein amyloid fibrillation. This study found that nitrogen-doped graphene (N-graphene) is the best in preventing the formation of harmful protein aggregates linked to PD, making it a promising option for treatment. This was so because N-graphene is the most effective at disrupting the structure of α-synuclein amyloids, showing strong interactions and causing significant instability compared to other monolayers (117, 118).

### 4.5 Metal-organic frameworks

The investigators have found that enzyme-integrated metal-organic frameworks (MOFs) had antioxidant activity and chiral-dependent BBB trans endocytosis. These frameworks may treat PD as anti-neuroinflammatory drugs (119). By adding extremely small platinum nanozymes (Ptzymes) to L-chiral and D-chiral imidazolate zeolite frameworks, chiral nanozymes are generated. In male PD mouse models, Ptzyme@D-ZIF accumulated more brain tissue than Ptzyme@L-ZIF. This was due to longer plasma presence and more BBB crossing routes, including clathrin- and caveolae-mediated endocytosis. These variables explain Ptzyme@D-ZIF’s increased therapeutic efficacy in reducing behavioural and pathological changes. Bioinformatics and biochemical studies show that Ptzyme@D-ZIF prevents neuroinflammation-induced apoptosis and ferroptosis (120) in damaged neurons. In enzyme-integrated chiral ZIF platforms, live organisms, metabolism, and therapeutic effects vary, suggesting the potential for PD medicines (121).

### 4.6 Mechanisms of action of NPs

The therapeutic effect of the NPs could be exerted on the PD through various processes. One basic feature is enhancing the drug delivery across the BBB, which ensures that a higher concentration of the therapeutic agent reaches the brain (122). This particular feature has an important role in PD as several drugs have limited ability to cross the BBB on their own (123). Besides this, NPs could also provide a controlled and sustained release of drugs, which maintains the therapeutic levels over extended periods and reduces side effects involved with peak-dose fluctuations. In addition to this, NPs can be designed to target specific cellular pathways associated with PD pathology. It has been found that customized NPs could deliver antioxidants or anti-inflammatory agents to lower the OS and neuroinflammation, which are the major contributors to neuronal degeneration. Besides this, NPs could also be used to deliver neurotrophic factors or GT agents. The neurotrophic factors or GT agents promote neuronal survival and repair, which offers potential disease-modifying effects (124). Table 5 exhibits a summarized form of the implications of nanomedicine on PD.

**Table 5:** A summary of the implication of nanomedicines on PD.

Nanoparticles	ROA	Outcome of study	References
L-dopa/dopamine (DPA)	Intracranial	Implantation of DOPA-loaded NPs in the frontal lobe of mice, surgically resulting in constant dispersion till 30 days with low systemic scape	(208)
	NA	In the brains of rats, nano silica-DOPA reservoirs surgically implanted showed circling behaviour by apomorphine	(209)
	Intraperitoneal injection (IPI)	Successful delivery of dopamine-loaded CNP through BBB; concentration peak around 80 min	(210)
	NA	The study performed *in vitro* exchange of NPs conjugated with galactose and DOPA by an artificial membrane through GLUT-1 receptors	(211)
	Subcutaneous injection	NPs combined with L-dopa and benserazide showed dyskinesias	(13)
	Intranasal	NPs + L-dopa in a gel resulted in brain recovery	(212)
	NA	Zinc and aluminum-based NPs combined with L-dopa showed the potential for releasing the drug without any toxic effects in a sustained manner	(213, 214)
	Intranasal	Release of combined nano-dopa particles exhibited improved results; motor coordination increased twofold after 28 days compared to conventional treatment	(215)
		Association of L-dopa and CNTs showed a sustained release pattern *in vitro*, with less toxicity to PC12 cells	(216)
	Intranasal	Delivered dopamine NPs to brain; elevated dopamine volume was found in the striatum after repeated administration, with no evident tissue damage	(217)
	IV infusion	Dopamine PLGA-NPs in rats exhibited successful cross of BBB, resulting in increased and impulsive locomotor functions and significantly lower rotational scores	(206)
Ropinirole (RPN)	Transdermal	Transdermal nanocarrier + RPN generated twice boosted bioavailability in comparison to free drug transdermal approaches	(218)
	NA	RPNs associated with NPs had better bioavailability and controlled release patterns *in vitro*. Close to 98% penetration to *ex-vivo* sheep mucosa	(219)
	Intranasal	Delivery of RPN NPs had a constant release pattern, resulting in reduced tremors in rats compared to RPN alone	(220, 221)
	NA	RPN chitosan NPs released to rats had better bioavailability than free drug solution	(222)
	NA	Delivery of chitosan-coated RPN nanoemulsion; enhanced muscular coordination and swimming in mice	(223)
Bromocriptine (BrP)	Intraperitoneal	In rats, BrP SLNs have a faster onset and longer half-life than free BrP, stabilizing plasmatic levels	(224)
	Intraperitoneal	Encapsulated BrP is used in rats, resulting in attenuating motor deficits	(225)
	Intraperitoneal	BrP chitosan NPs were observed to improve absorption and brain targeting, depicting a better future as a nano-drug	(226)
Apomorphine		Apomorphine-loaded nanobubbles have the potential to penetrate artificially, proven in successful release patterns	(227)
	Oral	In the brains of rats apomorphine, SLNs were delivered, and an observed high increase in bioavailability resulted in an improved diseased state	(228)
Urocortin	Intranasal	Urocortin with odorranalectin-NPs prolonged nasal residence time, increasing intranasal absorption. Rotational movement induced by apomorphine reduction	(229)
Rasagiline	Intranasal	Rasagiline chitosan NPs used intranasally in mice were observed to have better bioavailability than single-drug or intravenous NPs	(230)
Selegiline		Selegiline HCl-loaded-chitosan NPs prepared and tested *in vitro*, presenting a controlled-release profile up to 1 day and 4 h	(231)

It could be concluded that nanomedicine exhibits hope for treating PD by using several modes of administration, for instance, intracranial implantation, intraperitoneal injection, transdermal, intranasal, and oral delivery. Several NPs like L-dopa/dopamine, ropinirole, bromocriptine, apomorphine, urocortin, odorranalectin, rasagiline, and selegiline HCl have shown improved DD, bioavailability, controlled release, and symptom management in PD therapy.

## 5 Targeting and delivery strategies of NPs for PD

For the successful treatment of PD by NPs, there are two important features: firstly, effective targeting and delivery. From the various pieces of literature, it has been observed that numerous investigations have developed strategies to enhance the specificity and efficiency of NPs delivery to the brain and the affected neurons (22, 125).

### 5.1 Surface functionalization

The NPs mediated enhanced drug delivery across the BBB and improved diagnostic accuracy. NPs could be achieved by source functionalization by specific molecules Numerous investigations have demonstrated that NPs could be functionalized with targeting ligands (antibodies, peptides, or small molecules). These targeting ligand surface-functionalized NPs bind specifically to receptors or proteins expressed on the dopaminergic neurons’ surface. Such targeted strategies ensure that the therapeutic agents are directly delivered to the damaged cells. Additionally, these targeted approaches reduce unwanted side effects and improve therapeutic outcomes (125). Moreover, functionalized NPs can act as contrast agents in imaging techniques, selectively accumulating in affected brain areas to enhance disease detection and monitoring (126). In one of the studies, the investigator functionalized selenium NPs (SeNPs) with polyvinylpyrrolidone (PVP) and polysorbate 20, which demonstrated potential in enhancing dopamine and L-dopa delivery across the BBB. The study demonstrated efficient internalization and improved pharmacokinetic profiles (127). Moreover, a study involved functionalized AuNPs for targeting α-synuclein, which enhanced diagnostic accuracy by improving signal detection in biological samples and enabled early and precise diagnosis (104). In a study carried out by Huang et al. (128), exenatide-modified deferoxamine NPs were developed to target the brain’s lesion areas. This approach combined iron chelation and inflammatory suppression to upgrade neurological deficits in PD mice.

### 5.2 Penetration of BBB

One of the major hurdles in treating PD is overcoming the BBB, which can be efficiently achieved by surface modification of the NPs. Such surface-functionalized NPs could enhance their ability to cross the BBB through receptor-mediated transcytosis, which may more efficiently penetrate the brain. In addition, NPs delivery could be facilitated by external stimuli, like magnetic fields or ultrasound. Furthermore, NPs can be engineered to mimic endogenous transport mechanisms, like those used by nutrients or hormones, to cross the BBB more effectively (22). In one of the studies, Wu et al. (129) used Lpc-BoSA/CSO (functionalized lipid NPs) to increase the BBB permeability and facilitate the delivery of peptide drugs like exenatide. These NPs significantly enhance dopamine levels and reduce α-synuclein expression, addressing symptoms and disease progression. Polymeric NPs have been functionalized with the ligands that target BBB transporters. This enhanced the permeability and efficacy in treating neurological diseases, including PD (130).

### 5.3 Controlled release

Specifically, engineered NPs could help to achieve the controlled drug release from NPs under specific stimuli, like variation in the pH, temperature, or the presence of specific enzymes, which could trigger. The engineered NPs may ensure the drug is released at the desired site and time, demonstrating enhanced therapeutic efficacy and reduced side effects. In one of the studies, the investigator developed various organic NPs for the encapsulation and sustained release of puerarin to improve brain delivery for treating PD. The engineered NPs extend their *in vivo* half-life and improve their bioavailability and accumulation in the brain to treat the symptoms of PD. Compared to the puerarin alone, puerarin-NPs showed significantly improved cellular internalization, permeation, and neuroprotective effects (131).

## 6 Nanocarrier systems

The use of NPs in the biomedical field is continuously increasing. Nanomedicine is a promising domain for developing more effective and targeted treatment approaches for PD. The NPs used to deliver the drug to the targeted sites are called nanocarriers. The nanocarrier systems, like liposomes, nanogels, dendrimers, and SLNPs, have been proven effective for treating PD.

### 6.1 Liposomes

**Figure 9:**
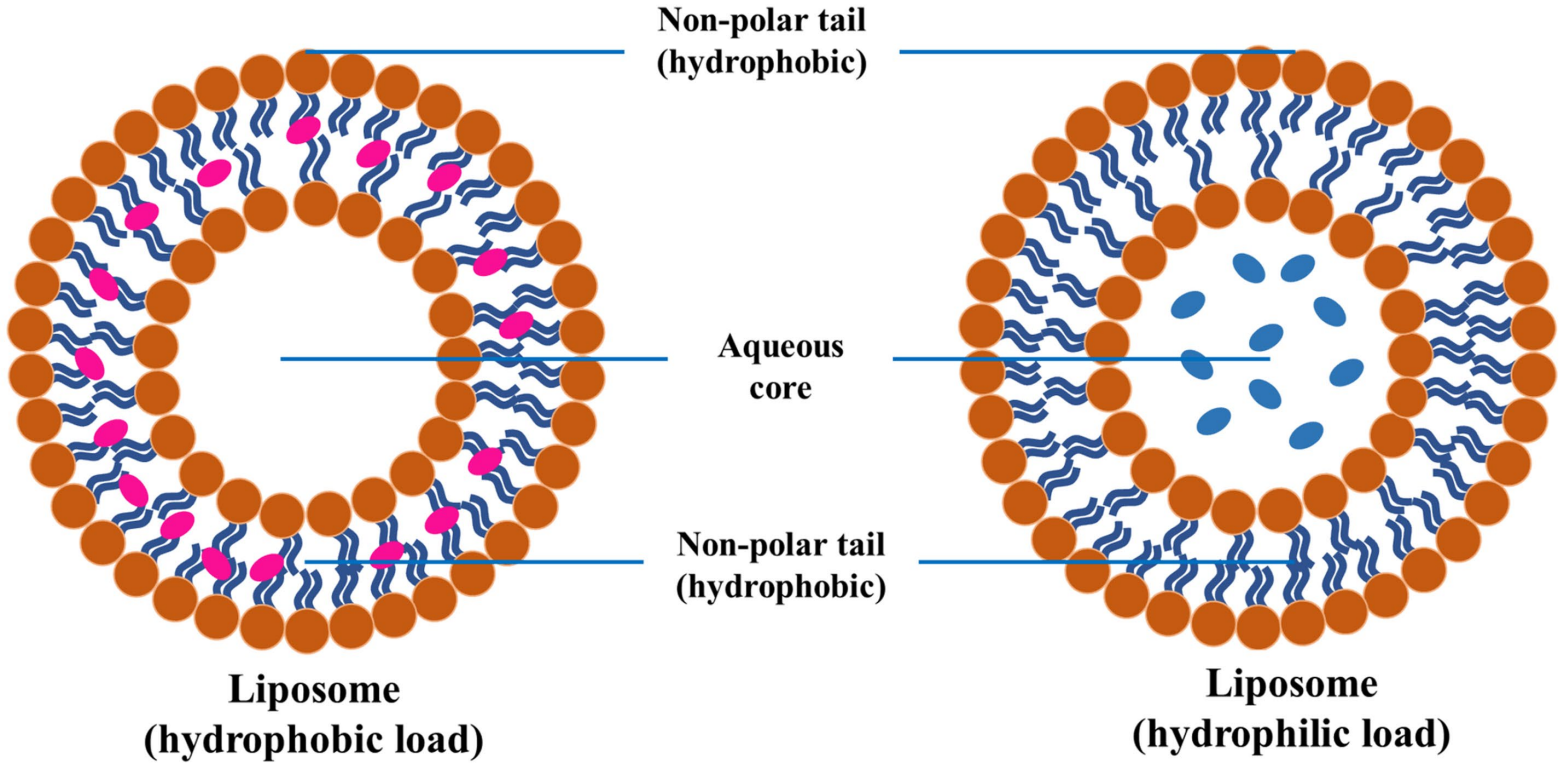
Schematic diagram of liposome and encapsulation of hydrophilic and hydrophobic molecules, reproduced from Jagaran et al. (145) with permission.

Liposomes are spherical vesicles comprising phospholipid bilayers that can encapsulate hydrophilic and hydrophobic drugs (Figure 9). Liposomes offer significant advantages as drug delivery systems for PD, particularly due to their ability to encapsulate various types of drugs and their potential for targeted delivery to specific cells or tissues, such as dopaminergic neurons (132, 133). The surface modification of the liposome with ligands (antibodies or peptides) ensures targeted delivery to dopaminergic neurons, which could improve their targeting capabilities and efficacy and reduce side effects. In addition to this, PEGylation (attachment of polyethylene glycol chains) has been employed for longer circulation time and reduced immunogenicity. The liposomes could be engineered for the PD drug’s effective, controlled, and sustained release. Various external factors could trigger the release of drugs from these engineered liposomes, for instance, variation in the pH or enzymatic activity (134–136).

### 6.2 Nanogels

Nanogels have also gained significant attraction in nanomedicine as nanocarriers. These three-dimensional, hydrophilic polymer networks could swell up in aqueous environments. As a drug delivery system, nano gels have several advantageous features for the PD, like high drug loading capacity, biocompatible nature, and safeguarding the encapsulated drugs from degradation (137, 138). Currently, investigators are emphasizing the development of smart nanogels that respond to environmental stimuli, like temperature, pH, or magnetic fields, to acquire controlled and site-specific release of drugs. Investigators have developed temperature-responsive nano gels that could release the drugs loaded on them at elevated temperatures found in inflamed or diseased tissues. This ensures that the drug is delivered precisely where it is needed in the brain. Moreover, nanogels could also be functionalized with targeting ligands to increase their specificity for dopaminergic neurons. Several reports report the formulation of multifunctional nano gels that combine drug delivery with imaging capabilities, which helps in enabling real-time monitoring of PD treatment efficacy (139).

### 6.3 Dendrimers

Dendrimers are organic molecules characterized by their highly branched, tree-like polymeric structure, including well-defined architectures and many functional groups on their surfaces (140). The distinctive design of dendrimers allows for exact regulation of size, shape, and surface functioning. These characteristics render them an attractive contender for drug delivery applications. Dendrimers may be designed for the targeted delivery of drugs, genes, or other therapeutic agents to the brain to treat PD (141). Recent investigations indicate the development of dendrimers with targeting moieties capable of selectively traversing the BBB and binding to dopaminergic neurons. Furthermore, dendrimers can be engineered to release encapsulated pharmaceuticals reacting to particular stimuli, similar to liposomes. The regulated release of the medicine from the dendrimer guarantees that the drug is delivered exclusively to the target location, minimizing systemic adverse effects. Currently, investigators are examining the capability of dendrimers to concurrently deliver various therapeutic drugs, perhaps providing a comprehensive strategy for PD treatment (142, 143).

### 6.4 Solid lipid nanoparticles

**Figure 10:**
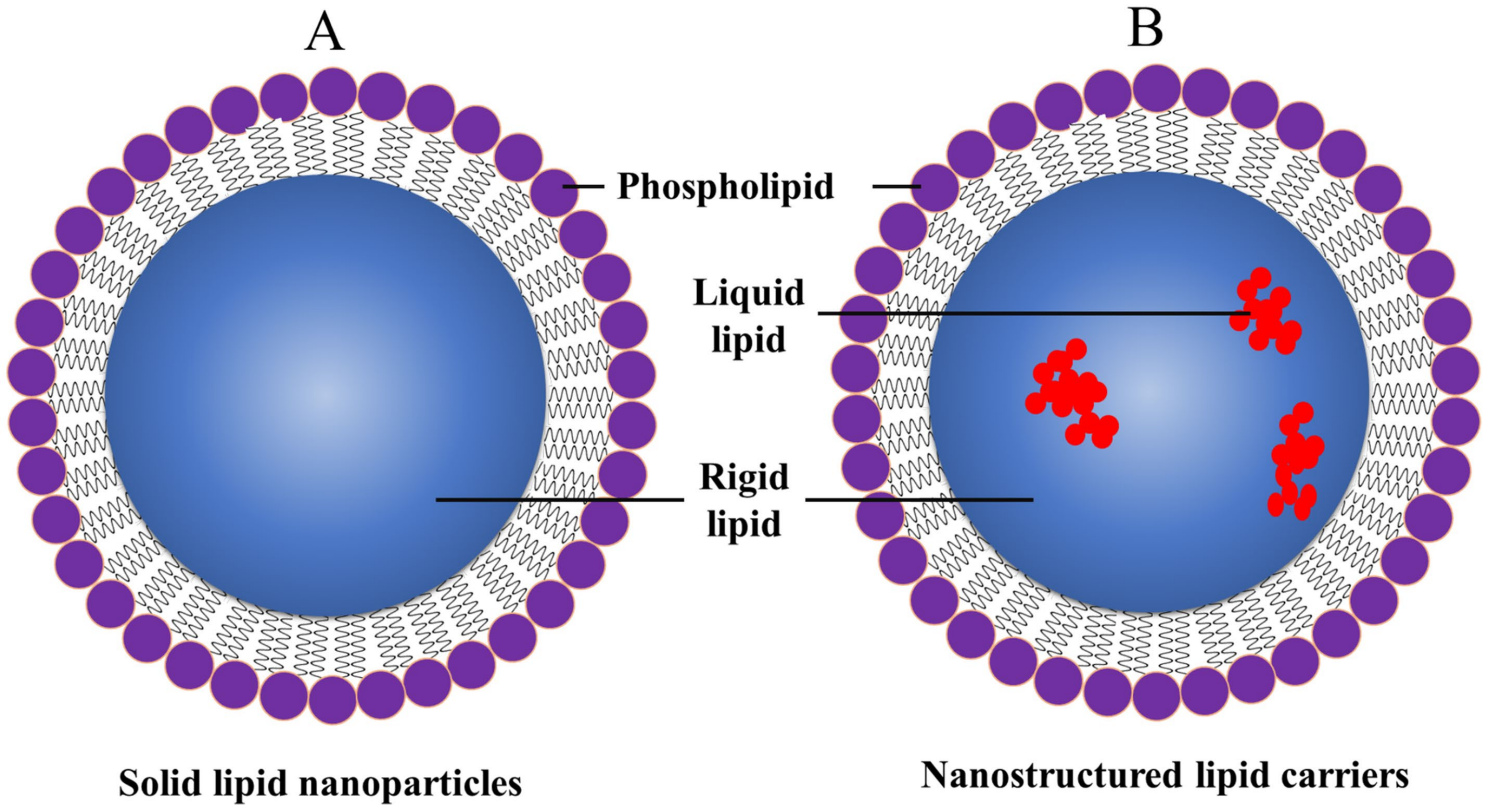
General structure of **(A)** SLNPs and **(B)** nanostructured lipid carriers (NLCs), reproduced from Jagaran et al. (145) with permission.

SLNs are submicron-sized particles of solid lipids that could encapsulate lipophilic and hydrophilic drugs (Figure 10). Such SLNs offer numerous advantages, like controlled drug release, biocompatibility, and protecting the encapsulated drugs from degradation (97, 144). Investigators have reported improving the stability of SLNs, drug loading capacity, and targeting capabilities. SLNs could be functionalized by targeting ligands to improve their specificity for dopaminergic neurons in treating PD. Moreover, SLNs could also be tailored like dendrimers and liposomes to ensure the crossing through the BBB, which remains a major challenge for neurologists who treat PD (97). The engineered SLNs ensure that the loaded therapeutic agents reach the specific sites of the brain for the treatment of PD. Nowadays, several investigators are exploring the role of SLNs in delivering neuroprotective agents, antioxidants, and anti-inflammatory drugs. Such types of SLNs offer the potential to modify the progression of PD and improve patient outcomes. Besides, this investigation is also going on the co-delivery of multiple drugs using SLNs, which will provide a synergistic approach for the treatment of PD (24).

## 7 Gene therapy and nanomedicine

GT focuses on introducing, removing, or altering the genetic material within a patient’s cells, which offers a potential disease-modifying strategy. With the combination of GT and nanomedicine, GT could attain more precise and effective delivery of genes to target cells. Several investigations focus on the application of GT for the treatment of PD, which focuses on gene delivery systems, CRISPR/Cas9 technology and NPs, and clinical applications and challenges (145, 146).

For effective GT, robust drug delivery systems are required to transport therapeutic genes to specific cells and tissues. The NP-based innovative methods may provide several advantages over traditional viral vectors. These advantages may include lower immunogenicity, higher loading capacity, and the potential to be engineered for targeted delivery and controlled release (147).

Lipid-based NPs (LBNPs), like liposomes and lipid nanoparticles (LNPs), are widely used to deliver genes. Such LBNPs could encapsulate the gene and protect it from enzymatic degradation. This could facilitate their entry into cells. Moreover, LBNPs could be surface-modified with certain ligands targeting specific dopaminergic neuron receptors. This will further enhance the precision of gene delivery in PD (148). Various investigations have found that polymeric NPs could be a potential gene delivery system. Such polymeric NPs encapsulate the plasmid DNA, siRNA, or mRNA, protect them from enzymatic degradation, and offer sustained release. The specificity of the polymeric NPs for the neuron cells could be further enhanced by functionalization with the targeting moieties (148, 149).

Several NPs have been designed to mimic the structure and function of viral vectors called viral mimetic NPs. These viral mimetic NPs do not have immunogenicity and could efficiently deliver genetic material to the brain by exploiting natural cellular uptake processes. It is possible to achieve targeted delivery to specific neuronal populations by increasing the therapeutic outcomes of PD (146).

One of the most emerging techniques is the CRISPR/Cas9 system, which has revolutionized gene editing by permitting precise modifications to the genome. Delivering CRISPR/Cas9 components into specific cells, specifically neurons, poses significant challenges. Such NPs provide a versatile and efficient delivery platform.

In recent investigations, LNPs have demonstrated a huge potential in delivering CRISPR/Cas9 components to target cells. These LNPs could encapsulate the Cas9 protein and guide RNA (gRNA) within LNPs. LNPs protected these components from enzymatic degradation and facilitated their uptake by cells. The specificity of the LNPs for dopaminergic neurons could be enhanced by surface functionalization with targeting ligands. This may enable precise gene editing by using CRISPR/Cas9 in PD (150).

Investigators are exploring polymeric NPs’ capability to deliver CRISPR/Cas9. Like LNPs, polymeric NPs also encapsulate the Cas9 and gRNA. Due to this encapsulation, the CRISPR/Cas9 gene will be protected from enzymatic degradation and may provide sustained release of the gene. In addition to this, polymeric NPs could be surface-modified with targeting moieties. The surface modification of the polymeric NPs facilitates the targeted delivery of the CRISPR/Cas9 components to neurons damaged by PD. This approach could reduce unwanted side effects and enhance therapeutic efficacy (151, 152).

The combination of GT and nanomedicine holds valuable promise for treating PD. Various preclinical studies have shown the feasibility and efficacy of utilizing NPs for gene delivery in PD models. GT could deliver neuroprotective genes to promote neuronal survival and function. For instance, delivering genes that encode neurotrophic factors like GDNF (glial cell line-derived neurotrophic factor) could support dopaminergic neurons and slow down the progression of the disease. NPs provide a targeted and efficient delivery system for these therapeutic genes, ensuring they reach the affected neurons in the brain. The combination of CRISPR/Cas9 technology with NPs provides the potential to correct genetic mutations associated with PD. Some investigators are utilizing CRISPR/Cas9 to target and edit mutations in the LRRK2 gene, which are implicated in familial forms of PD. NPs allow precise delivery of the CRISPR/Cas9 components to neurons, allowing for accurate gene editing and potential disease modification (153).

Even though the combination of GT and nanomedicine has gained several advancements in the PD treatment field, there are still various challenges in the clinical application. Such a combination ensures efficient and targeted delivery of genetic material to the brain by overcoming BBB. Moreover, the long-term expression of therapeutic genes in the brain remains a major challenging task. Moreover, there is a requirement for a thorough evaluation of safety and immunogenicity for all the NP-based gene delivery systems during the clinical trials. The researchers are focusing on optimizing the NPs design, improving targeting strategies, and developing new materials for improved efficiency of delivery and safety. Integrating advanced imaging techniques and biomarkers could also help monitor therapeutic outcomes. Besides this, such integrated techniques could guide the development of personalized GT strategies for PD (154).

## 8 Nanomedicine for neuroprotection

Nanomedicine offers promising strategies for neuroprotection in PD treatment by utilizing NPs to deliver neuroprotective agents across the BBB. These NPs can enhance drug bioavailability, target specific brain regions, and minimize side effects, making them a cutting-edge approach to managing neurodegenerative diseases. Recent advancements in nanomedicine offer promising strategies for neuroprotection, which aims to preserve and restore neuronal function. The various nanomedicines demonstrating neuroprotective effects are antioxidant NPs, anti-inflammatory NPs, and neurotrophic factor delivery for neuroprotection in PD. All these types of neuroprotective NPs are described briefly below.

### 8.1 Antioxidant nanoparticles

The OS is characterized by an imbalance between the generation of ROS and the brain’s antioxidant defenses, which plays a crucial role in the pathogenesis of PD. Excessive ROS could damage the cellular components, leading to neuronal death. Antioxidant NPs are engineered particles that can effectively scavenge ROS in biological systems. These NPs are designed to deliver antioxidants more effectively to target sites, enhancing their therapeutic potential. The integration of antioxidants into NPs improves their bioavailability and allows for targeted delivery, reducing side effects and increasing efficacy. Investigators are developing antioxidant NPs like nanoceria, polymeric antioxidant NPs, selenium NPs (Se-NPs), etc., to combat OS and protect neurons (155). Antioxidant NPs have emerged as a promising therapeutic strategy for the treatment of PD, primarily due to their ability to target OS and neuroinflammation, which are key pathological features of the disease. These NPs can enhance drug delivery across the BBB, improve pharmacokinetic profiles, and provide neuroprotective effects.

#### 8.1.1 Cerium oxide NPs (nanoceria)

Cerium oxide NPs (nanoceria) possess intrinsic antioxidant features as they have the potential to switch between Ce^3+^ and Ce^4+^ oxidation states, mimicking the activity of natural antioxidant enzymes. Recent investigations exhibited that nanoceria can scavenge ROS, lower OS, and protect dopaminergic neurons in PD models. The functionalization of nanoceria with targeting ligands enhances their delivery to the brain and enhances their neuroprotective efficacy (156). Dillion et al. (157), reported that PD is strongly associated with OS, and CeONPs act as a free radical scavenger. The investigator has hypothesized that CeONPs may be a promising disorder-modifying therapy for PD treatment. The investigator for the first reported that the CeONPs preserve striatal dopamine and safeguard the dopaminergic neurons in the substantia nigra in the MPTP-mouse model of PD. Finally, the investigator concluded CeONPs might be a potential novel nano pharmaceutical for neurodegenerative disorder therapy.

Gao et al. (158) developed the (Q@CeBG nanoreactor, which combines CeO_2_ nanozyme and quercetin) and has shown potential in reducing OS and polarizing microglia from a pro-inflammatory to an anti-inflammatory state. This is achieved through focused ultrasound to enhance BBB penetration, demonstrating significant neuroprotection and immunomodulatory effects in PD models.

#### 8.1.2 Polymeric antioxidant nanoparticles

The investigation has revealed that the polymeric NPs could be loaded with several antioxidant compounds (curcumin, resveratrol, or N-acetylcysteine). Such NPs provide a sustained release of antioxidants, which ensures prolonged protection against OS. By maximizing their neuroprotective effects, surface functionalization with targeting ligands can further improve their specificity for dopaminergic neurons.

El Nebrisi (159) emphasized the processes underlying curcumin’s neuroprotective properties as a prospective treatment for patients with PD. Curcumin (turmeric) is a polyphenolic compound with potent anti-inflammatory, mitochondrial protecting, antioxidant, free radical scavenging and Fe-chelating effects. It acts as a promising therapeutic and nutraceutical agent for treating PD. The immune system’s “cholinergic anti-inflammatory pathway” is mostly regulated by stimulating nicotinic receptors and, more specifically, selective α7 nicotinic acetylcholine receptors (α7-nAChR). Recently, α7-nAChR has been suggested as a possible therapy strategy for PD.

Wang et al. (160) used a systematic review to evaluate the efficacy of curcumin in experimental PD comprehensively. The investigators used electronic and manual searches for the literature and identified studies describing curcumin’s efficacy in animal PD models. The investigation revealed that 13 studies with 298 animals showed that curcumin helps protect neurons and reduce inflammation in PD, although one study found it ineffective against certain disruptions in the brain. The study concluded that curcumin showed a marked efficacy in the experimental model of PD. This suggests curcumin is probably a neuroprotective drug for human PD patients.

El Nebrisi et al. (161) demonstrated that curcumin protects neurons in a 6-hydroxydopamine (6-OHDA) rat model of PD by functioning via α7-nAChR. The researchers conducted *in vivo* experiments to evaluate the influence of α7-nAChR on curcumin’s protective effects in a rat PD model. Rats were subjected to intrastriatal injections of 6-hydroxydopamine (6-OHDA). The results demonstrated that the intragastric administration of 200 mg/kg of curcumin considerably enhanced abnormal motor behaviour. Immunoreactivity of tyrosine hydroxylase (TH) in the substantia nigra and caudoputamen suggested its neuroprotective role against the degeneration of dopaminergic neurons. The α7-nAChR-selective antagonist methyllycaconitine (1 μg/kg) delivered intraperitoneally nullified the neuroprotective effects of curcumin on animal behaviour and TH immunoreactivity.

Numerous studies have demonstrated the strong neuroprotective and antioxidant properties of polyphenols like curcumin through enhanced activity of detoxification systems such as glutathione peroxidase, catalase, and superoxide dismutase (SOD). Several studies have revealed that curcumin modulates epigenetics in neurological diseases and neuroinflammation (162, 163). However, curcumin-mediated epigenetic modulation’s neuroprotective effects are yet unclear (164). Tripathi and Bhawana (165) reviewed mitochondrial dysfunction, epigenetic modulations, and mitoepigenetics in age-associated neurological diseases. It also examined curcumin’s epigenetic machinery-regulated neuroprotective benefits in treating and preventing neurodegenerative disorders.

Another natural polyphenol with neuroprotective properties is resveratrol (RES) due to its antioxidant property and ability to protect mitochondria. Increased ROS production induces OS, which damages cells. Lipid peroxidation, oxidative protein modification, and DNA damage may ensue. Multiple PD model researchers reported that RES pretreatment boosts endogenous antioxidant status and directly scavenges ROS to reduce OS. Much research has studied the role of RES in modulating Nrf2 in PD models, which recognise oxidants and modulate antioxidant defence. Zamanian et al. (166) studied the impacts of RES activity in both *in vitro* and *in vivo* models of PD, as well as the molecular mechanisms behind it. The collected information demonstrated that by upregulating Nrf2 and decreasing OS, RES therapy offers neuroprotection against PD. Furthermore, the investigators focused on providing scientific evidence for the neuroprotective qualities of RES against PD and outlined the mechanism underlying therapeutic development considerations.

Castellani et al. (97), have developed SLNs co-loaded with dopamine for intranasal administration, which offers a multifunctional approach to PD treatment. These NPs demonstrate sustained release of dopamine and high cytocompatibility, showing potential in reducing oxidative cellular damage and providing neuroprotection.

#### 8.1.3 Selenium nanoparticles

Se-NPs have been used as an antioxidant for various therapeutic purposes. Literature has proven that both types of Se-NP functionalized and non-functionalized, can protect mitochondria by increasing the levels of ROS-scavenging enzymes in the brain. It has been reported that Se-NPs can also promote the synthesis of dopamine. Se-NPs can lower cellular toxicities by inhibiting the aggregation of tau, α-synuclein, and/or Aβ. The ability of the BBB to absorb Se-NPs, which maintain a healthy microenvironment, is essential for the homeostasis of the brain. Recently, Umapathy et al. (167) highlighted the importance of Se-NPs on stress-induced neurodegeneration and its critical control. The investigator suggested that due to the potential of Se-NPs to inhibit cellular stress and the pathophysiology of PD, they can act as a promising neuroprotector with anti-inflammatory, non-toxic, and antimicrobial features.

Kalčec et al. (127), functionalized SeNPs with polyvinylpyrrolidone and polysorbate 20 to enhance drug delivery across the BBB and act as antioxidants. They demonstrate efficient internalization and improved permeability for dopamine and L-dopa, highlighting their potential as drug-delivery vehicles in PD treatment.

### 8.2 Anti-inflammatory nanoparticles

Neuroinflammation is characterized by the activation of microglia and the release of pro-inflammatory cytokines, contributing to the progression of PD. Anti-inflammatory NPs can be engineered to deliver anti-inflammatory agents directly to affected sites, minimizing systemic side effects and enhancing therapeutic efficacy. Anti-inflammatory NPs are being developed to modulate the inflammatory response and protect neurons from inflammatory damage (168).

#### 8.2.1 Dexamethasone-loaded NPs

Dexamethasone is a potent anti-inflammatory steroid that can modulate neuroinflammation, a critical factor in PD progression (169). The encapsulation of dexamethasone (a potent anti-inflammatory corticosteroid) in NPs could be achieved by improving its delivery to the brain and decreasing systemic side effects. Some recent investigations have demonstrated that dexamethasone-loaded NPs can effectively cross the BBB and may further reduce neuroinflammation and safeguard the dopaminergic neurons in PD models. Such NPs could be easily engineered for controlled release by ensuring sustained anti-inflammatory effects (170). The encapsulation of dexamethasone in PLGA NPs allows for controlled release and targeted delivery, reducing systemic side effects and enhancing efficacy (169).

#### 8.2.2 Nanoparticles targeting inflammatory pathways

NPs targeting inflammatory pathways offer a promising approach to managing PD by addressing neuroinflammation and OS, which are critical in the disease’s progression. These NPs are designed to cross the BBB and target specific inflammatory pathways, thereby mitigating the effects of neuroinflammation and promoting neuronal health (171). NPs could be tailored to target specific inflammatory pathways involved in PD. One of the investigations reported that NPs were loaded with nuclear factor kappa B (NF-κB) inhibitors, a key regulator of inflammation, which could suppress the microglial activation and decrease the production of pro-inflammatory cytokines. The NF-κB is a key regulator of inflammation. These targeted strategies can potentially modulate neuroinflammation more precisely and effectively by reducing the off-target effects (172). In one study, it was observed that engineered extracellular vesicle-based nanoformulations (EVNs) target neuroinflammation by blocking peripheral inflammatory cell infiltration and promoting the Nrf2–GPX4 pathway to suppress inflammation (173). Liu et al. (174), engineered the NPs to cross the BBB using specific ligands or surface modifications. For instance, nanocapsules modified with Angiopep-2 enhance brain delivery by targeting the BBB, while cRGD targets integrin receptors upregulated in PD-affected areas. Jiang et al. (175) developed chiral metal-organic frameworks with nanozymes, demonstrating chiral-dependent BBB transendocytosis, with Ptzyme@D-ZIF showing higher brain accumulation due to multiple endocytosis pathways.

Nanoparticles often incorporate ROS-scavenging materials to reduce OS, a key factor in neuroinflammation. For example, Pt nanozymes in a dual synergetic nanoreactor exhibit enhanced antioxidative activities, mitigating oxidative damage and promoting microglia polarization to an anti-inflammatory phenotype (176).

There are various synergistic approaches for treating PD by targeting inflammatory pathways by the NPs. For instance, carrier-free nanocapsules deliver dopamine and catalase to the PD brain by combining dopamine replacement with neuroinflammation mitigation. This addresses both dopamine depletion and neuroinflammation. This approach enhances dopaminergic signaling and reduces neuroinflammation, slowing disease progression (174). Furthermore, Fan et al. (176) demonstrated that nanoreactors combining dihydroquercetin and Pt enzymes show synergistic effects in reducing OS and controlling the inflammatory microenvironment, thus preventing neuronal damage.

### 8.3 Neurotrophic factor delivery

Neurotrophic factors are crucial proteins that support neurons’ growth, survival, and differentiation. They play a significant role in developing and maintaining the nervous system by preventing apoptosis and promoting neuronal regeneration. These proteins, including brain-derived neurotrophic factor (BDNF), nerve growth factor (NGF), and neurotrophin-3, are involved in various cellular processes that ensure the proper functioning of neurons. From several pieces of literature, it has been observed that the delivery of neurotrophic factors to the brain holds a promising strategy for neuroprotection and neurorestoration in PD. In addition, NPs act as an effective delivery system for these neurotrophic factors, ensuring their stability and bioavailability (172).

#### 8.3.1 Glial cell line-derived neurotrophic factor-loaded NPs

GDNF-loaded NPs have been explored as a potential treatment for PD due to GDNF’s neuroprotective effects on dopaminergic neurons. GDNF supports dopaminergic neurons and plays a role in neural development and repair, but its delivery across the BBB remains a challenge (177). NPs could potentially address these issues by enhancing the delivery and stability of GDNF in the brain. The encapsulation of the GDNF by the NPs protects them from being degraded from enzymatic attack and facilitates delivery to the brain. One recent investigation showed that GDNF-loaded NPs could significantly improve dopaminergic neuron survival and motor function and minimize neurodegeneration in PD models (178).

Some major drawbacks associated with the clinical application of such NPs are their poor ability to cross the BBB and the inconsistent clinical efficacy of GDNF in PD treatment. Clinical trials have shown that GDNF does not significantly improve motor symptoms in PD patients and is associated with a higher incidence of serious adverse events (179). Long-term exposure to GDNF can lead to dephosphorylation of key signalling pathways, reducing its neuroprotective effects. This suggests the need for optimized delivery methods.

#### 8.3.2 Brain-derived neurotrophic factor-loaded NPs

BDNF is a critical protein belonging to the neurotrophin family, playing a significant role in neuronal growth, synaptic plasticity, and overall brain health (180). It regulates axonal and dendritic growth, synaptic plasticity, and long-term potentiation, essential for learning and memory. BDNF’s influence extends to various psychiatric and neurological disorders, making it a focal point for therapeutic research and potential interventions. BDNF promotes the survival and maturation of dopaminergic neurons, which are crucial in PD pathology. NPs can cross the BBB and deliver the BDNF to the brain, thereby increasing the bioavailability of BDNF in the brain. Due to this, there is sustained release and targeted delivery to dopaminergic neurons (181).

The delivery of BDNF via NPs could potentially slow disease progression and improve motor function. The preclinical studies have shown promising results in animal models, where NP-mediated delivery of BDNF has led to neuroprotective effects (182). Boyton et al. (122) have shown that by encapsulating BDNF, NPs can provide controlled and sustained release, which may enhance the therapeutic efficacy and reduce the frequency of administration. Moreover, the surface modification of the NPs with targeting ligands can further improve the specificity and efficacy of BDNF delivery, which promotes the survival and function of the neurons (183). Table 6 summarizes all the nanoparticles used for neuroprotection in PD treatment.

**Table 6:** Summary of all the nanoparticles used for neuroprotection in Parkinson’s disease treatment.

Nanoparticles	Types of neuroprotectant	MOA	Role	References
MgO-based polydopamine (anti-SNCA plasmid, puerarin)	Antioxidant	Antioxidation, anti-inflammation, inhibition of α-synuclein accumulation	Targets deep tissues, activates under NIR light, penetrates BBB	(232)
Cerium oxide NPs (nanoceria)	Antioxidant	Mimic natural antioxidant enzymes by switching oxidation states (Ce^3+^/Ce^4+^), functionalized for targeted brain delivery	Scavenge ROS, lower OS, and safeguard dopaminergic neurons	(156, 157)
Polymeric antioxidant NPs	Antioxidant	Provide sustained release of antioxidants like curcumin, resveratrol, and N-acetylcysteine	Target dopaminergic neurons, reduce inflammation, and protect against OS	(159–161)
Mannitol-modified PLGA (dihydroquercetin, Pt nanozymes)	Antioxidant	Antioxidative activities, anti-inflammatory effects	Mitigates oxidative damage, promotes microglia polarization	(176)
Selenium NPs (Se-NPs)	Antioxidant	Increase ROS-scavenging enzymes, promote dopamine synthesis, inhibit aggregation of tau and α-synuclein	Protect mitochondria, enhance brain homeostasis, and reduce neurodegeneration	(127)
Dexamethasone-loaded NPs	Anti-inflammatory	Cross BBB for controlled release of dexamethasone, reducing systemic side effects	Modulate neuroinflammation and safeguard dopaminergic neurons	(169, 173)
Fe-Cur NCPs (curcumin)	Anti-inflammatory	Scavenging radicals, suppressing neuroinflammation	Alleviates OS, mitochondrial dysfunction, inflammatory conditions	(233)
NPs targeting inflammatory pathways		Block peripheral inflammatory cell infiltration, target NF-κB pathways, and promote Nrf2-GPX4 pathway activation	Address neuroinflammation and OS and enhance neuronal health	(172)
GDNF-loaded NPs	Neurotrophic	Encapsulate GDNF to protect it from enzymatic degradation and enhance brain delivery	Improve dopaminergic neuron survival and motor function, reduce neurodegeneration	(177, 178)
pGDNF DNA NPs	Neurotrophic	Induces transgene expression, protects dopamine neurons	Non-invasive GT enhances neuroprotection	(234)
BDNF-loaded NPs	Neurotrophic	Encapsulate BDNF for controlled release and enhance delivery to dopaminergic neurons	Promote neuronal growth, synaptic plasticity, and brain health; slow PD progression	(180, 182)
GMO NPs	Curcumin, piperine	Augmented inhibition of α-synuclein oligomers and fibrils	Enhances bioavailability, penetrates BBB, anti-parkinsonism effect	(235)

## 9 Advantages and disadvantages of the nanotechnology vs. existing approaches

Nanotechnology offers promising advancements in treating PD by addressing existing therapies’ limitations, such as poor bioavailability and inability to halt disease progression (184). Traditional treatments like dopamine replacement and DBS primarily manage symptoms without altering the disease course. Through innovative drug delivery systems, nanotechnology aims to enhance therapeutic efficacy and reduce side effects. The major advantages of nanotechnology in PD treatment include enhanced drug delivery, neuroprotection and neuro repair, versatility and functionalization, and reduced side effects (185).

The major disadvantages of nanotechnology include safety and long-term effects, complexity and cost and regulatory challenges (186–188). Furthermore, the potential toxicity of NPs in the treatment of PD is a significant concern, especially when considering long-term use or large doses. These NPs may cause neurotoxic effects. Several studies have shown the dual nature of nanoparticles, where their beneficial properties are often accompanied by potential risks, particularly in the context of neurodegenerative diseases like PD. It has been found that SiO_2_ NPs can exacerbate PD pathology by promoting α-synuclein aggregation, mitochondrial impairment, OS, and neuronal apoptosis. These effects were observed in both *in vitro* and *in vivo* models, indicating a strong potential for SiO_2_ NPs to initiate and develop PD-like symptoms (74). Metallic NPs, can induce neurotoxicity through mechanisms such as OS, autophagy dysfunction, and activation of harmful signaling pathways. NPs can penetrate the CNS and potentially cause neuroinflammation and neurodegeneration. The mechanisms of NP-induced neurotoxicity include the generation of free radicals, immune responses, and neuroinflammation, which can lead to cell necrosis and other adverse effects. The healthcare industry is concerned with the regulatory aspects of NP use in neuronal disorders. There is a need for comprehensive risk assessments and safety regulations to mitigate adverse reactions in humans and animals (58).

## 10 Conclusion

Exploring nanomedicine for treating Parkinson’s disease presents a promising and transformative approach. Nanoparticles offer several advantages over traditional therapies, including improved drug delivery, targeted therapeutic interventions, and crossing biological barriers like the BBB. The application of various types of nanoparticles, such as lipid-based, polymeric, and metallic NPs, has demonstrated potential in effectively delivering therapeutic agents to the affected brain regions. Furthermore, integrating GT and CRISPR/Cas9 technology with nanomedicine opens new avenues for addressing the underlying causes of neurodegeneration in PD. This combination promises precise and efficient delivery of genetic material to specific neurons, potentially correcting genetic mutations and promoting neuroprotection. Despite these advancements, some challenges must be addressed, including optimising NP design, ensuring long-term expression of therapeutic genes, and thoroughly evaluating safety and immunogenicity. The future of PD treatment lies in the successful translation of nanomedicine from the laboratory to clinical practice, paving the way for personalized and effective treatment strategies. Overall, nanomedicine represents a significant leap forward in managing Parkinson’s disease, offering hope for improved symptom management, enhanced patient outcomes, and the potential to slow disease progression. Continued research and development in this field are essential to thoroughly understand its potential and bring about a new era in neurological care.
